# RAD51-mediated R-loop formation acts to repair transcription-associated DNA breaks driving antigenic variation in *Trypanosoma brucei*

**DOI:** 10.1073/pnas.2309306120

**Published:** 2023-11-21

**Authors:** Mark John Girasol, Marija Krasilnikova, Catarina A. Marques, Jeziel D. Damasceno, Craig Lapsley, Leandro Lemgruber, Richard Burchmore, Dario Beraldi, Ross Carruthers, Emma M. Briggs, Richard McCulloch

**Affiliations:** ^a^College of Medical, Veterinary and Life Sciences, School of Infection and Immunity, Wellcome Centre for Integrative Parasitology, University of Glasgow, Glasgow G12 8TA, United Kingdom; ^b^Faculty of the MD-PhD in Molecular Medicine Program, College of Medicine, University of the Philippines Manila, Manila 1000, Philippines; ^c^College of Medical, Veterinary and Life Sciences, School of Cancer Sciences, University of Glasgow, Glasgow G12 0YN, United Kingdom; ^d^Institute for Immunology and Infection Research, School of Biological Sciences, University of Edinburgh, Edinburgh EH9 3FL, United Kingdom

**Keywords:** trypanosome, antigenic variation, DNA repair, recombination, RNA–DNA hybrid

## Abstract

RNA–DNA hybrids are epigenetic features of all genomes that intersect with many processes, including transcription, telomere homeostasis, and centromere function. Increasing evidence suggests that RNA–DNA hybrids can provide two conflicting roles in the maintenance and transmission of genomes: They can be the triggers of DNA damage, leading to genome change, or can aid the DNA repair processes needed to respond to DNA lesions. Evasion of host immunity by African trypanosomes, such as *Trypanosoma brucei*, relies on targeted recombination of silent Variant Surface Glycoprotein (*VSG*) genes into a specialized telomeric locus that directs transcription of just one *VSG* from thousands. How such *VSG* recombination is targeted and initiated is unclear. Here, we show that a key enzyme of *T. brucei* homologous recombination, RAD51, interacts with RNA–DNA hybrids. In addition, we show that RNA–DNA hybrids display a genome-wide colocalization with DNA breaks and that this relationship is impaired by mutation of RAD51. Finally, we show that RAD51 acts to repair highly abundant, localised DNA breaks at the single transcribed *VSG* and that mutation of RAD51 alters RNA–DNA hybrid abundance at 70 bp repeats both around the transcribed *VSG* and across the silent *VSG* archive. This work reveals a widespread, generalised role for RNA–DNA hybrids in directing RAD51 activity during recombination and uncovers a specialised application of this interplay during targeted DNA break repair needed for the critical *T. brucei* immune evasion reaction of antigenic variation.

## Significance

RNA–DNA hybrids aid DNA damage repair in ways that remain unclear. RAD51-directed recombination of silent variant surface glycoprotein (*VSG*) genes allows *Trypanosoma brucei* to evade host immunity. How such *VSG* switching is targeted to the single transcribed *VSG* and how recombination is initiated are unknown. We find that *T. brucei* RAD51 interacts with RNA–DNA hybrids and describe a genome-wide, RAD51-directed correspondence between hybrids and DNA break locations. *VSG* switching exploits this DNA break repair function of RNA–DNA hybrids, since a pronounced break localises to the transcribed *VSG* and RAD51 loss increases break abundance and results in loss of associated RNA–DNA hybrids. Thus, *T. brucei* uses a specialised application of RNA–DNA hybrid formation during DNA damage repair for sustaining host infections.

Rapid change in genetic content or organisation, either in localised regions or across the genome, is integral to the life of many organisms. Examples of localised genome variation in eukaryotes include yeast mating-type switching (1, 2) and immunity gene rearrangements to allow the expression of T cell receptors and B cell antibodies (3). Variation encompassing larger parts of genomes include chromosome fragmentation during the development of ciliates (4)and chromosome and gene copy number variation during the life cycle of *Leishmania* (5, 6). A myriad of strategies induces such changes, including the generation of DNA breaks by sequence-specific endonucleases, locus-directed replication stalling, and base modification. In most cases, the DNA lesions generated are repaired to effect genetic change, normally by harnessing generalised DNA repair pathways, including homologous recombination (HR), nonhomologous end-joining, and microhomology-mediated end-joining. Antigenic variation is a widespread immune evasion strategy in which surface antigens are periodically switched to escape elimination of the infecting pathogen by host adaptive immunity (7). Though some such antigen switches can be catalyzed by transcriptional control mechanisms (8), locus-targeted antigen gene rearrangement occurs in many different bacteria, fungi, and protists (9, 10). Perhaps surprisingly, the processes that initiate antigen gene recombination are poorly characterised in all but *Neisseria gonorrhoeae* (11, 12), *Trypanosoma brucei* (see below), and to some extent, *Borrelia burgdorferi* (13, 14).

Antigenic variation in *T. brucei* relies upon continuous changes in the identity of the variant surface glycoprotein (*VSG*) expressed on the parasite cell surface (15). A single *VSG* gene is transcribed, by RNA Polymerase (Pol) I, in a parasite cell from one of around 15 telomeric loci termed bloodstream expression sites (BESs) (16, 17). In these complex multigene transcription sites, a *VSG* appears always to be the most telomere-proximal gene and is separated from a variable number of upstream expression site associated genes (*ESAG*s) (18) by an array of 70-bp repeats. Such repeats are also found in smaller numbers upstream of thousands of silent *VSG* genes and pseudogenes that are mainly located in arrays in the subtelomeres of 11 megabase-sized chromosomes (19–24). *T. brucei* can activate a silent *VSG* using HR, most commonly by gene conversion (25), where the donor *VSG* sequence is copied into the actively transcribed BES and replaces the previously expressed *VSG*. The 70-bp repeats frequently demarcate the upstream extent of *VSG* gene conversion. *VSG* switching can also occur by turning off complete transcription at the single transcribed BES and turning on complete transcription from one of the other previously silent BES. The mechanisms behind transcriptional switching remain elusive, but there is no evidence this process involves recombination. *VSG* switching is impaired when the key enzyme of HR, RAD51, is mutated (25–28), an effect mirrored after mutation of other factors that aid homology-directed strand exchange by HR, including BRCA2 (29, 30) and at least one RAD51-related protein (paralogue) (31, 32).

How HR is targeted to the active BES to cause recombinational activation of a silent *VSG* is an unresolved question (33). Engineering the targeting of yeast I-SceI endonuclease activity within the BES in the vicinity of the *VSG* elicits *VSG* recombination (27, 34, 35), demonstrating that a DNA double-strand break (DSB) can be a trigger of *VSG* switching. However, no native BES-focused nuclease has been described, and though ligation-mediated PCR has detected breaks in the BESs, this assay has not revealed evidence for a discrete break location within, or indeed limited to, the active BES (27, 28, 34). Loss of telomere length or protection can also result in BES breaks and *VSG* switching (28, 36, 37). Finally, mutation of the RNA–DNA endonucleases RNase H1 or RNase H2 leads to *VSG* switching and BES damage (38–40), effects also seen after loss of the DNA damage signalling kinase ATR (41). One potential explanation to connect RNA–DNA hybrids and *VSG* switching is the demonstration that the actively transcribed BES is replicated earlier than all silent BESs (42, 43), but several aspects of any model that might explain the roles of RNA–DNA hybrids are unclear. One key question is how RNA–DNA hybrids contribute to *VSG* recombination switching; are they the cause of DNA breaks in the BES, or do they form in response to breaks? RNA–DNA hybrids are widespread epigenetic features of all genomes and can be resolved by RNase H-mediated degradation of the RNA within the hybrids (44). R-loops are a particular form of RNA–DNA hybrids in which RNA base-pairs with one strand of the DNA helix, displacing the other DNA strand. R-loops can be generated during transcription, but further activities are emerging in all genomes, including initiation and arrest of DNA replication (45, 46), transcription activation and termination (47, 48), chromatin formation (49, 50), and telomere function (36, 51). In many such activities, R-loops can lead to genome instability (52–54), at least in part by generating DNA breaks, such as during class switch recombination in mammalian B lymphocytes (55, 56). However, it is also increasingly clear that RNA–DNA hybrids can form in response to DNA DSBs (57–59), though it is unclear in what circumstances they inhibit or promote repair of such lesions by HR.

Here, we demonstrate that *T. brucei* RAD51 interacts with RNA–DNA hybrids and that loss of the recombinase causes genome-wide changes in R-loop abundance. Moreover, we demonstrate widespread colocalisation of R-loops with DNA DSBs, a relationship that is impaired by RAD51 loss. Finally, we demonstrate that RAD51 is needed to repair highly abundant and localised DNA breaks at the *VSG* in the single active BES, and that loss of RAD51 alters R-loop distribution both within the BES and across the silent *VSG* archive. Our data therefore reveal both generalised roles for R-loops in RAD51-directed HR in *T. brucei* and uncover specialised features of this activity in the critical immune evasion reaction of antigenic variation.

## Results

### *T. brucei* RAD51 Binds RNA–DNA Hybrids.

**Fig. 1.**
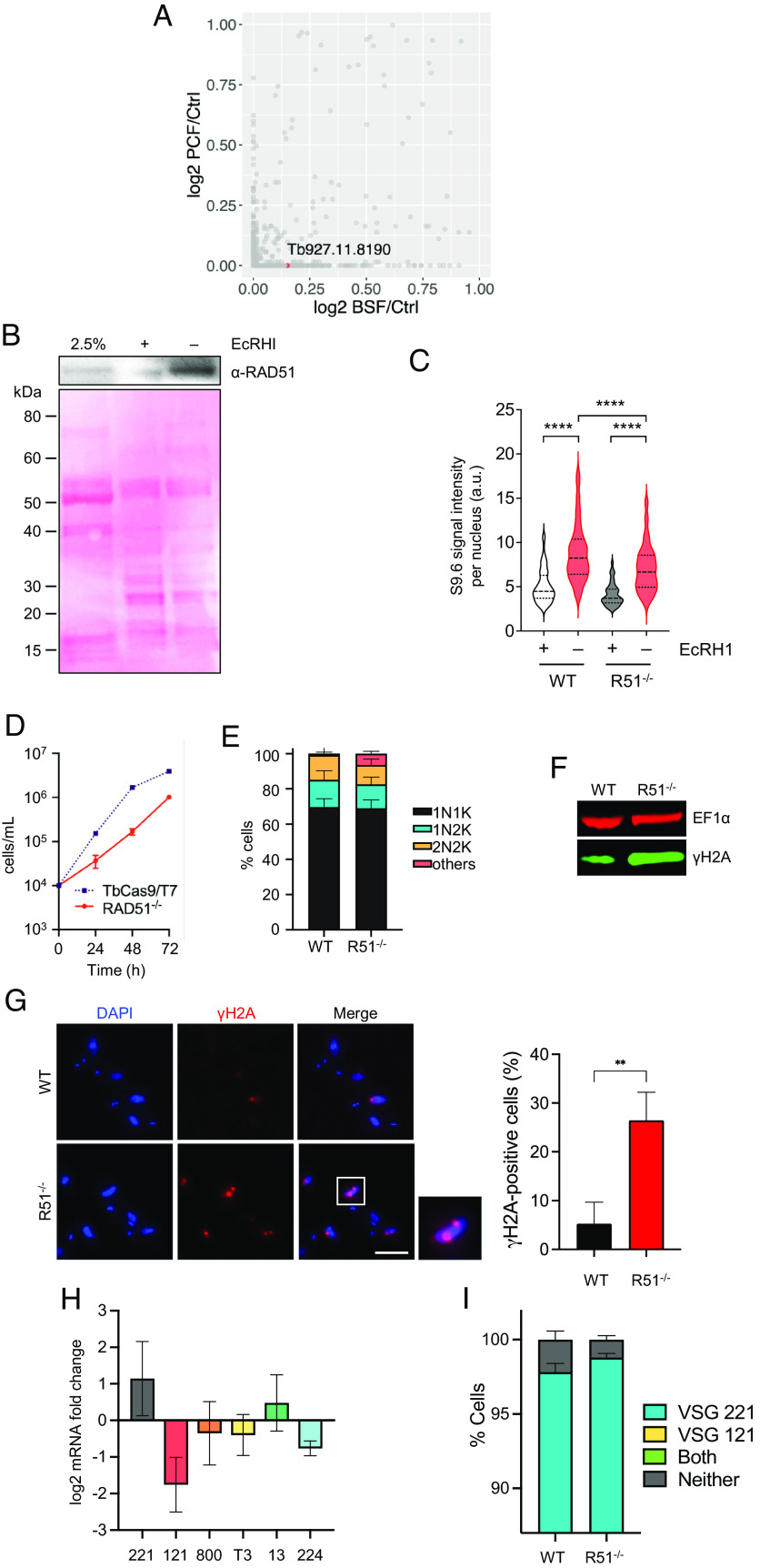
*T. brucei* RAD51, which promotes *VSG* switching, interacts with RNA–DNA hybrids. (*A*) Scatter plot of log_2_-transformed mean emPAI values of gene MS data identified from BSF and PCF S9.6 DRIPs relative to benzonase-treated controls; *RAD51* (gene ID Tb927.11.8190) is highlighted. (*B*) *Top* panel shows western blot of RAD51 in *RNaseH1–/–* cells (input) and after S9.6 DRIP, with (+) and without (–) EcRHI treatment; *Bottom* panel shows the same blot stained with Ponceau. (*C*) Violin plots show intensity of nuclear S9.6 immunofluorescence signal in WT and *rad51*–/– cells, with (+) and without (–) EcRHI treatment, in each case for >100 cells; the median is shown by a heavily dotted line and the interquartile range by surrounding lightly dotted lines; statistical significance was determined through one-way ANOVA followed by Šídák’s multiple comparisons test: ****P < 0.0001. (*D*) Growth comparison of *rad51–/–* and WT (TbCas9/T7 expressing) cells; error bars represent SD from three independent experiments. (*E*) Cell cycle profiles of *rad51–*/– and WT cells as determined by DAPI staining of nucleus (N) and kinetoplast (K) configurations in individual cells: 1N1K, 1N2K, 2N2K, or aberrant (others); values are shown as a proportion of >300 cells, and error bars represent SD from three independent experiments. (*F*) γH2A western blot of *rad51*–/– and WT whole-cell extracts; EF1α was used as a loading control. (*G*) Representative microscopy images of γH2A immunofluorescence of *rad51–/–* mutants and WT cells (scale bar, 5 µm); graph shows the percent of cells with detectable γH2A signal (error bars signify SD from three independent experiments, counting at least 50 cells in each experiment; statistical significance determined using *t* test, **P < 0.01). (*H*) RT-qPCR quantification of different *VSG* mRNA levels in *rad51*–/– mutants compared to WT cells; error bars indicate SD of two independent experiments. (*I*) Graphical representation of *VSG* immunofluorescence analysis showing the proportion of *rad51*–/– and WT cells expressing VSG221 alone (blue), VSG121 alone (yellow, no cells detected), both VSG221 and VSG121 (green, no cells detected), or neither (gray); error bars represent SD from three independent experiments, counting >300 cells in each experiment.

Our previous work on *T. brucei* RNase H1 and RNase H2 revealed a connection between R-loops in the *VSG* BESs and damage-induced *VSG* switching (38, 39). To test and explore this connection, we performed DNA-RNA hybrid immunoprecipitation (DRIP) with S9.6 antiserum from nuclear extracts of both bloodstream form (BSF, mammalian host stage) and procyclic form (PCF, insect vector stage) *T. brucei* and identified interacting proteins by mass-spectrometry (MS) (60, 61). Then, 602 proteins were recovered in at least one of six DRIP-MS replicates from the two life cycle forms (Girasol et al., BIORXIV/2023/540366); amongst these putative RNA–DNA hybrid interactors, RAD51 was detected in DRIP-MS from BSF cells but not PCF cells (Fig. 1*A*). To test this predicted RAD51-R-loop interaction, we performed DRIP in an *RNase H1*–/– (null) mutant, where RNA–DNA hybrids could be more readily precipitated, and the recovered chromatin was separated and analysed by western blot with anti-RAD51 antiserum. RAD51 signal was more abundant in the DRIP sample than in the input, and the signal was diminished upon treatment with *Escherichia coli* RNase HI (EcRHI; Fig. 1*B*). To ask further if RAD51 acts upon RNA–DNA hybrids, we generated *rad51*–/– mutants (Fig. 1*C* and *SI Appendix*, Fig. S1*A*). To do so, we engineered MiTat1.2 BSF cells, which predominantly express VSG221 (also named VSG2) from BES1 (see below) (38, 39), to allow modification via CRISPR-Cas9 (62). We then compared S9.6 immunofluorescence in the Cas9-expressing parental cell line (hereafter called wild type, WT) and *rad51–/–* cells (*SI Appendix*, Fig. S1*B* and see below) and found that the signal was significantly reduced in the mutant (Fig. 1*C*). In both parasite lines, the S9.6 signal was depleted by treatment with EcRHI, confirming the antiserum mainly detects RNA–DNA hybrids in these conditions. These data reveal interaction of RAD51 with RNA–DNA hybrids and indicate a role for the recombinase in homeostasis of the levels of these nucleic acid structures.

### Loss of RAD51 Reduces *VSG* Switching.

To test whether *rad51–/–* cells generated by CRISPR-Cas9 are comparable to previously described mutants, we tested for several phenotypes. First, the *rad51–/–* mutants displayed a growth defect relative to their WT cells (Fig. 1*D*), consistent with previous reports (26). An increased proportion of cells with abnormal nucleus to kDNA (mitochondrial genome) ratios (“others”; Fig. 1*E*) was seen in the mutants, a perturbation that did not result from Cas9 expression. Loss of RAD51 also resulted in increased levels of nuclear DNA damage, assessed via the detection of phosphorylated histone H2A (yH2A) (63) both by western blot (Fig. 1*F*) and immunofluorescence (Fig. 1*G*).

Next, we tested whether loss of RAD51 affects *VSG* switching. In the Cas9 WT cell line here used, a small proportion of cells (2.18 ± 0.34%) switched off expression of *VSG221* (transcribed from BES1) when replicating in culture (Fig. 1*I*), as seen in other WT cells (38, 39, 41, 64, 65). We tested whether this low level of stochastic switching was affected in the *rad51–/–* parasites: RT-qPCR revealed increased numbers of cells in the population that were transcribing *VSG221*, with a concomitant reduction in cells expressing 4 of 5 *VSG*s located in predominantly silent BESs (Fig. 1*H*); in addition, immunofluorescence with antiserum against VSG221 and VSG121 (expressed from predominantly silent BES3) revealed fewer cells in the mutant that did not express either protein (1.19 ± 0.15%) (Fig. 1*I*) and hence fewer cells that had switched to a distinct, nonprobed, *VSG*. These data confirm previous observations, using distinct assays (26), that deletion of *RAD51* impairs *VSG* switching. Notably, the effects of RAD51 loss on *VSG* switching are distinct from the increased *VSG* switching (observed using the same assays) after loss of either RNase H1 or RNase H2A (38, 39). We have not attempted to document the type of residual *VSG* switching that occurs in these *rad51–/–* cells, but previous work indicted that *VSG* recombination could still occur in the absence of the recombinase, as well as transcriptional switching between the *VSG* BES (26).

### Loss of RAD51 Alters R-loop Distribution in the *T. brucei* Genome Core.

To ask whether R-loop localisation is altered by loss of RAD51, DRIP coupled with deep sequencing (DRIP-seq) was performed using WT cells, *rad51*–/– mutants, and *RNase H1*–/– mutants (39, 66), and the sequenced DNA in the recovered RNA–DNA hybrids was then mapped to the *T. brucei* genome. DRIP specificity was confirmed by mapping DRIP-seq reads from two independent experiments in *rad51–/–* mutants to the coding sequence (CDS) of all RNA Pol II-transcribed genes, in each case with and without treatment with EcRHI (*SI Appendix*, Fig. S2*A*). A decrease in the DRIP-seq signal within the CDS (discussed below) was seen in both replicates in EcRHI-treated vs. -untreated experiments (*SI Appendix*, Fig. S2*A*). In addition, qPCR of DRIP for selected loci confirmed EcRHI sensitivity (*SI Appendix*, Fig. S2 *B* and *C*).

**Fig. 2.**
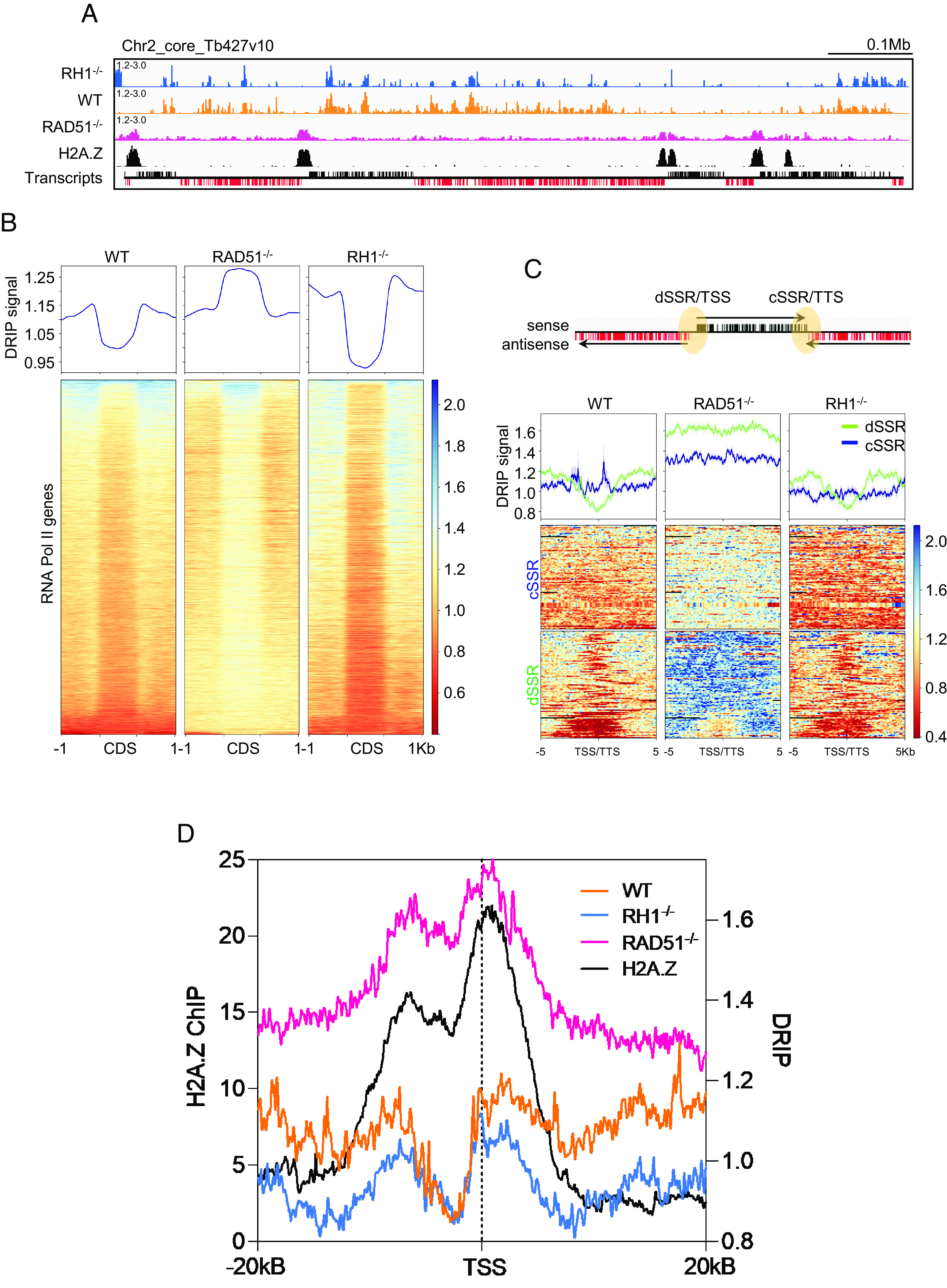
Loss of *T. brucei* RAD51 alters R-loop distribution within and at the TSSs of RNA Pol II multigene transcription units. (*A*) DRIP-seq read mapping (DRIP/input) across a selected region of chromosome 2 in *RNase H1–/–* (*RH1*–/–, blue), wild-type (WT, orange) and *rad51*–/– (magenta) cells, compared with H2A.Z ChIP-seq in WT cells (black); the bottom track shows genes with sense (black) and antisense (red) transcripts. (*B*, *Upper*) metaplot of DRIP-seq signal (relative to input) across all RNA Pol II–transcribed genes (scaled to the same size), including the 1 kb sequences that flank their CDS. (*Lower*) heatmap of DRIP-seq signal fold change across all CDS +/– 1 kb. (*C*) Metaplots (*Top*) and heatmaps (*Bottom*) of WT, *rad51*–/– and *RH1*–/– DRIP-seq signals across convergent (blue) and divergent (green) strand switch regions (cSSR and dSSR, respectively) between all RNA Pol II PTUs; the loci are aligned on their centres and include 5 kb of upstream and downstream sequence; the cartoon illustrates that cSSRs are TTSs, and dSSRs are TSSs. (*D*) Metaplots of H2A.Z ChIP-seq signal (black, left *y* axis) in WT cells is compared to DRIP-seq signal (right *y* axis) in WT (orange), *RH1*–/– (blue) and *rad51*–/– (magenta) (right *y* axis) cells over 40 kb regions surrounding all predicted TSSs. All metaplots show mean plus SEM (shaded).

We first examined the impact of RAD51 loss across the core genome that comprises multigenic (polycistronic) transcription units (PTUs). In previous work, we showed that R-loops are enriched at the start and ends of the PTUs and accumulate within the PTUs at sites of pre-mRNA editing (66). RAD51 loss had two clear effects associated with RNA Pol II transcription. First, the distribution of R-loops within the transcribed PTUs was altered in *rad51–/–* mutants (Fig. 2 *A* and *B*): in contrast to WT cells and *RNase H1–/–* mutants, where DRIP-seq signal was enriched in the flanks of CDSs, *rad51–/–* mutants showed greater levels of DRIP-seq enrichment within CDS (Fig. 2*B*; confirmed by DRIP-qPCR: *SI Appendix*, Fig. S2*C*). Thus, loss of RAD51 affects the localisation of R-loops at sites of trans-splicing and/or polyadenylation. Second, *rad51–/–* mutants displayed a marked accumulation of DRIP-seq signal at regions in WT cells that show enrichment for histone variant H2A.Z (Fig. 2*A*), a marker of PTU transcription start sites (TSSs) (67). Such enrichment was more pronounced than in WT cells or *RNase H1–/–* mutants (Fig. 2 *A* and *C*) and was not seen at transcription termination sites (TTSs). Comparing DRIP-seq pattern in the *rad51–/– mutants* relative to H2A.Z ChIP-seq in WT cells across all TSSs revealed considerable similarity (Fig. 2*D* and *SI Appendix*, Fig. S3*A*). Since the DRIP-seq signal was reduced by treatment with EcRHI (*SI Appendix*, Fig. S3*B*), we conclude that *T. brucei* RAD51 plays a hitherto undetected role in the deposition and/or regulation of R-loops associated with RNA Pol-II transcription initiation.

R-loops are also enriched at *T. brucei* centromeres, whose size and detailed sequence are uncertain (68, 69). We therefore used long-read Nanopore sequencing to resequence the Lister 427 genome, revealing 23 contigs that encompass at least part of a centromere (*SI Appendix*, Fig. S4). DRIP-seq mapping to these contigs confirmed R-loop localisation, with more pronounced signal in *RNase H1*–/– cells relative to WT in around 60% of the centromeres. In the *rad51–/–* mutants DRIP-seq signal was less pronounced in the centromeres that showed most enrichment in the *RNase H1*–/– mutants (*SI Appendix*, Fig. S4), indicating that here too the recombinase influences R-loop localisation.

### Loss of RAD51 Alters R-loop Distribution in the *VSG* Expression Sites.

**Fig. 3.**
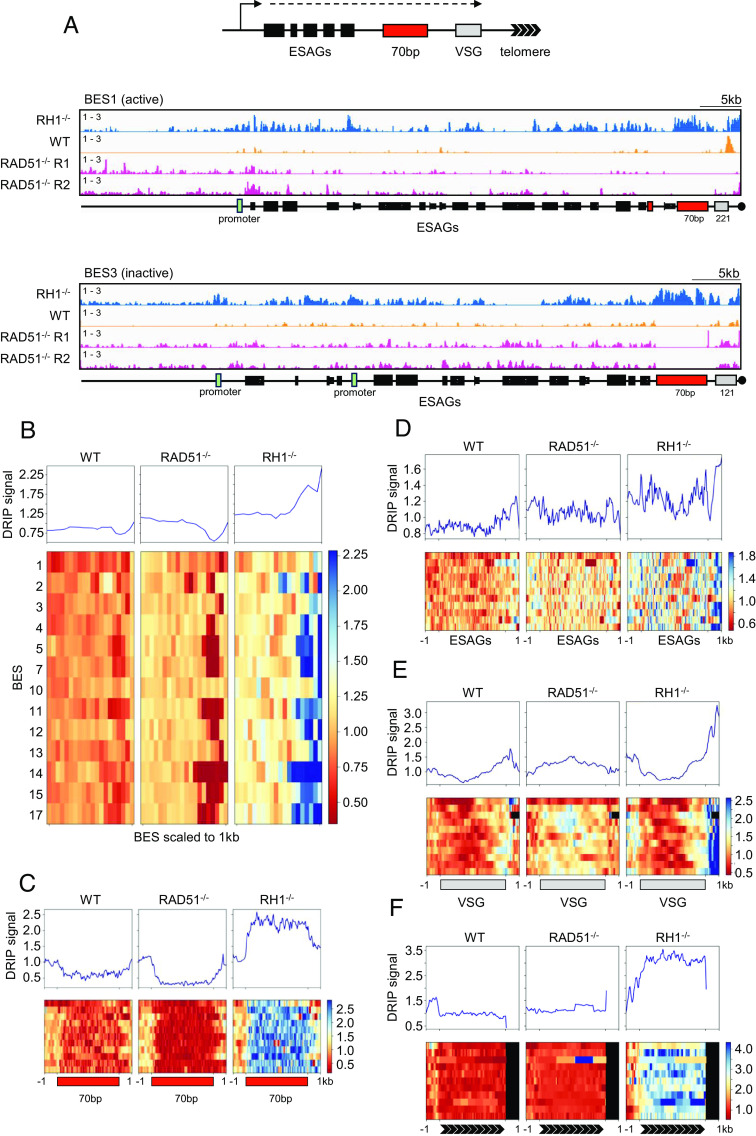
Loss of *T. brucei* RAD51 alters R-loop localisation in *VSG* expression sites. (*A*) A schematic representation of a BES is shown and, below, DRIP-seq read mapping (DRIP/input) across BES1 and BES3, comparing *RNase H1–/–* (*RH1–/–*, blue), WT (orange) and two independent *rad51*–/– (magenta) cells. Metaplots (*Top*) and heatmaps (*Bottom*) of WT, *rad51*–/– and *RH1*–/– DRIP-seq signals (relative to input) are shown for all BESs (*B*), or focusing on the 70-bp repeats (*C*), *ESAG*s (*D*), *VSG*s (*E*) and telomeres (*F*); in all cases, the BESs or BES features are scaled to the same size and data for silent BESs shows mean plus SEM (shaded).

We next examined DRIP-seq mapping in the *VSG* BESs (Fig. 3). Modest DRIP-seq enrichment was seen in silent or active BESs in WT cells, with somewhat greater signal detected downstream of the *VSG,* while DRIP-seq enrichment increased across the active and silent BESs in *RNase H1–/*– mutants (Fig. 3 *A* and *B*) (39). Loss of RAD51 had two effects on DRIP-seq distribution. First, and most pronounced, was reduced enrichment towards the telomere of the BESs (Fig. 3*B*) in *rad51–/–* cells, a change accounted for by reduced DRIP-seq signal across the 70-bp repeats, where DRIP-seq enrichment strikingly increased in the *RNase H1*–/– mutants (Fig. 3*C*) (39) relative to WT. Second, somewhat increased DRIP-seq enrichment was seen across the *ESAG*s upstream of the 70-bp repeats, though not to the same extent as in *RNase H1*–/– cells (Fig. 3*D* and *SI Appendix*, Fig. S2*B*). When comparing DRIP-seq mapping across the *VSG* genes in the BESs (Fig. 3*E*) or the telomeric repeats (Fig. 3*F*), any effects of RAD51 loss were less apparent than after RNase H1 loss, though R-loops were detectable in the *VSG*s (*SI Appendix*, Fig. S2*B*). Taken together, these data indicate that RAD51 is predominantly involved in the formation or stabilisation of R-loops at *VSG*-associated 70-bp repeats but not at telomeres, unlike TERRA in other eukaryotes (70, 71).

### Loss of RAD51 or RNase H1 Alters R-loop Distribution at Silent, Subtelomeric Array *VSG*s.

To ask whether RAD51’s influence on *VSG*-associated R-loops is limited to the *VSG* BESs, we next examined the subtelomeric *VSG* arrays, again using Nanopore long-read contigs, allowing us to assess R-loop localisation in this genome component for the first time (*SI Appendix*, Fig. S5). In WT cells, a similar distribution of DRIP-seq mapping to that observed around RNA Pol-II transcribed CDSs (Fig. 2*B*) was seen in the array *VSG*s (*SI Appendix*, Fig. S5 *A* and *B*), and this pattern became exaggerated in *RNase H1–/*– mutants. Thus, R-loops accumulate around potentially all subtelomeric *VSG* genes. Loss of RAD51 altered the R-loop distribution, with *rad51*–/*–* mutants showing greater DRIP-seq enrichment within the CDS than in the flanks (*SI Appendix*, Fig. S5 *A* and *B*), mirroring what was seen in RNA Pol-II transcribed genes in the genome core (Fig. 2 *A* and *B*). Together, these results indicate that *VSG*-associated R-loops, whose formation and/or stability is modulated by RAD51 and RNase H1, are not only seen in telomere-adjacent *VSG*s within transcription sites but are also widespread across the mainly silent subtelomeric *VSG* archive.

### DNA Breaks Mapped by BLISS (Breaks Labelling In Situ and Sequencing) Reveal Association with RAD51-Directed R-loops.

Given the above data reveals a link between R-loops and RAD51, we next sought to map the locations of DNA breaks in the *T. brucei* genome. To do so, we used BLISS, where DSBs in a cell population are mapped across the genome via adapter ligation, in vitro transcription, and RT-PCR amplification (72). A total of 17,134 and 11,160 BLISS “peaks” were predicted using MACS2 (73) in WT and *rad51–/*– populations, respectively, with similar distribution across a range of genomic landmarks (*SI Appendix*, Fig. S6*A*). These BLISS peaks represent sites of read accumulation on one DNA strand, meaning that they do not necessarily represent discrete two-ended DNA DSBs, and may instead be single ends of highly processed DSBs or one-ended DSBs (72, 74). In addition, 69% and 71% of BLISS reads were derived from the rRNA loci in WT and *rad51–/*– cells, respectively, suggesting that these are pronounced sites of DNA breaks, consistent with other organisms (75, 76). As the mapping removes reads of low quality, including from highly repeated sequences such as the rRNA, this abundance is not reflected in the genome-wide distribution plots (*SI Appendix*, Fig. S6*A*). Nevertheless, rRNA BLISS signal was more commonly found after loss of the recombinase (0.13% in WT, 0.25% in *rad51*–/*–*; *SI Appendix*, Fig. S6*A*), suggesting at least some rRNA gene breaks are acted upon by RAD51-directed HR. Metaplots showed that centromeres and tRNAs were flanked by BLISS peaks in both WT cells and *rad51–/*– mutants (*SI Appendix*, Fig. S6*B*). Most BLISS peaks were detected within the RNA Pol-II–transcribed PTUs (others, *SI Appendix*, Fig. S6*A*), where increased BLISS signal flanked the CDSs (*SI Appendix*, Fig. S6*B*). As this signal appeared less abundant in *rad51*–/*–* mutants, it may represent lesions repaired by means other than HR, such as transcription-coupled nucleotide excision repair (77). In contrast to other organisms, where DSBs are strongly associated with TSSs (78), we saw no clear BLISS signal localization at the start of the PTUs but did detect signal at TTSs in both WT and *rad51–/*– cells (*SI Appendix*, Fig. S6*C*). The three strongest BLISS peaks (*SI Appendix*, Fig. S6*D*) mapped to unitig851_maxicircle_Tb427v10, which represents mitochondrial maxicircles that are known to be found frequently as linear molecules after the generation of DNA DSBs due to topoisomerase II (79), and hence provide an example of correspondence between BLISS signal and known DNA breaks.

**Fig. 4.**
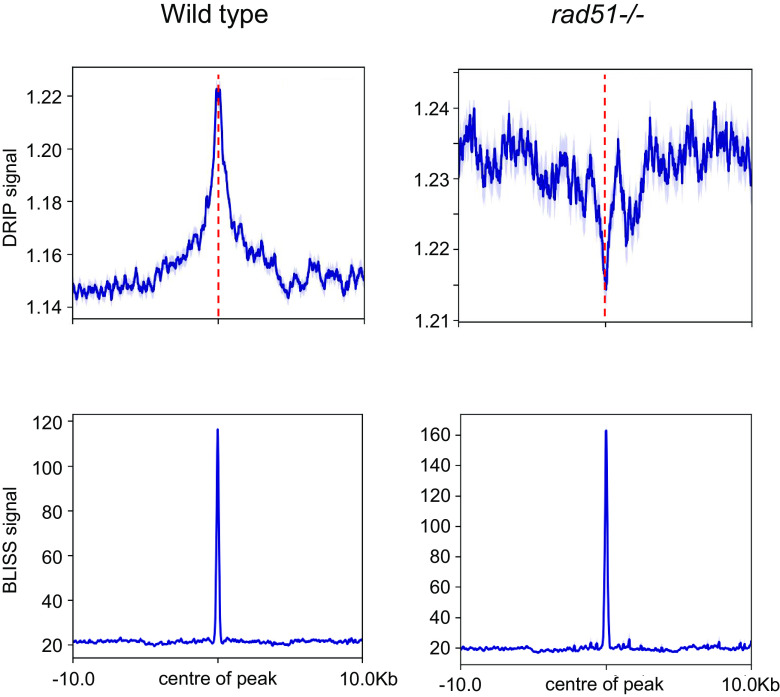
R-loop accumulation at DNA breaks is perturbed by loss of RAD51 across the *T. brucei* genome. Metaplots showing (*Upper*) the distribution of DRIP signal (DRIP/input) relative to (*Lower*) BLISS coverage (BLISS signal is read count per million mapped reads) in WT and *rad51–/*– cells. DRIP and BLISS signals are the mean plus SEM (shaded) aligned on the centre of the BLISS signal (dotted line) and including 10 kb of upstream and downstream sequence is shown.

To ask whether R-loops correlate with DSBs across the *T. brucei* genome, metaplots were generated for all loci where BLISS peaks were identified by MACS and were compared with DRIP-seq distribution at the same loci in both WT and *rad51*–/*–* cells (Fig. 4). DRIP-seq signal showed a pronounced enrichment that centred on the more discrete BLISS signal in WT cells, indicating break sites frequently associate with RNA–DNA hybrids. On the other hand, in *rad51–/*– mutants DRIP-seq enrichment was depleted at the centre of the BLISS signals. This global association was also seen at specific genome features (*SI Appendix*, Fig. S7). These data suggest that loss of RAD51 impairs R-loop localisation at BLISS-mapped breaks, indicating the recombinase acts to recruit or stabilize the hybrids at many DNA break sites, potentially during DSB repair.

### The Transcribed *VSG* Expression Site Contains a *VSG*-Localised DNA Break Acted upon by RAD51.

**Fig. 5.**
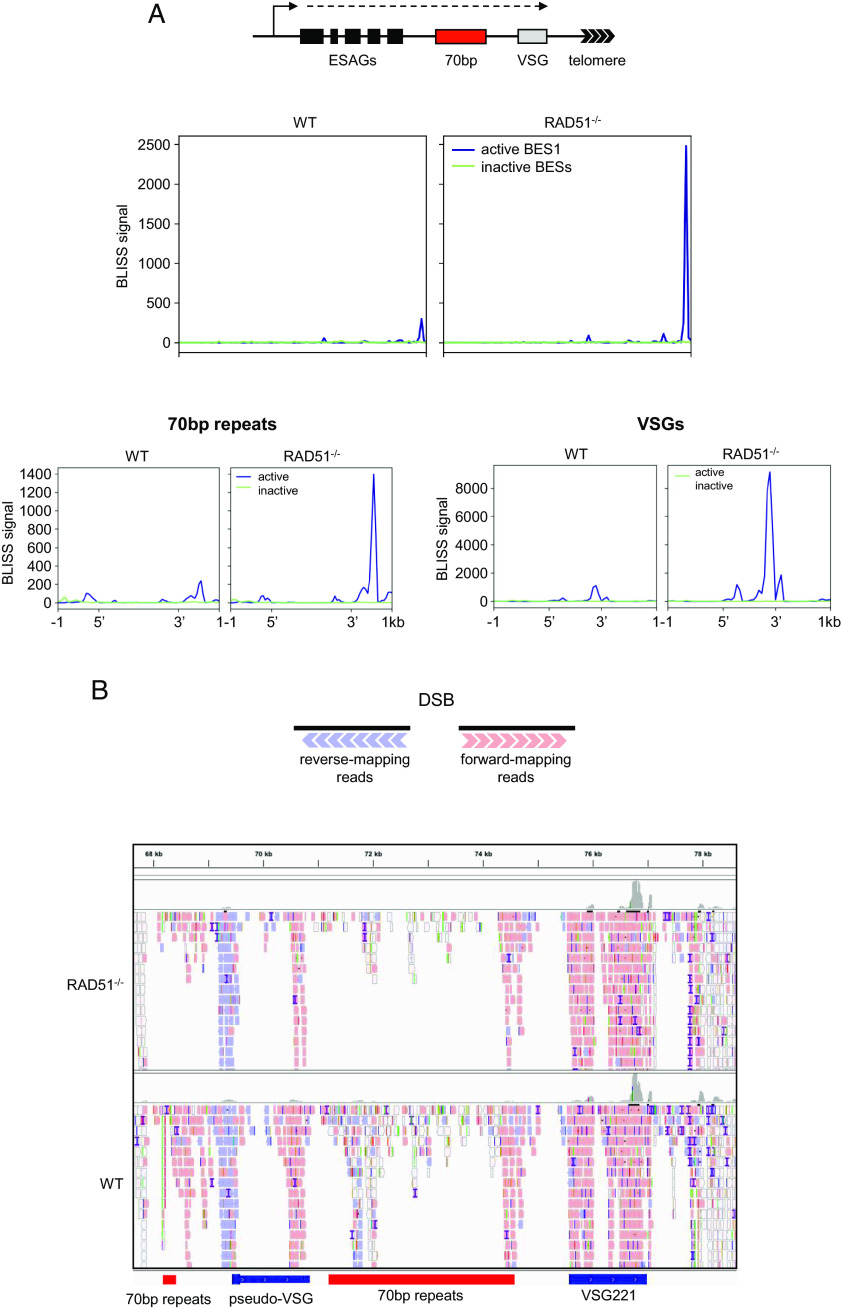
Pronounced DNA breaks at the 3′ end of the actively expressed *VSG* are repaired by RAD51. (*A*) Metaplots comparing the distribution of BLISS reads mapped to the active (blue, BES1) and all inactive (green) *VSG* expression sites (BESs; mean, plus SEM shaded) in WT and *rad51*–/*–* cells; BLISS signal is read count per million mapped reads (data are shown as averaged, normalised signal across the features of interest). A schematic of a BES is shown and BLISS mapping across the whole length of the BESs is shown in the main panel, while the *Lower* panels show BLISS signal across the 70-bp repeats (*Left*) and BES-housed *VSG*s (*Right*); in all cases the loci are scaled to be the same size. (*B*) The schematic shows the predicted orientation of mapped BLISS reads around a discrete DSB, and the figure below details the distribution of forward- and reverse-mapping reads at the telomere-proximal region of active BES1 in both WT and *rad51–/*– cells; locations of *VSG221*, a *VSG* pseudogene and 70-bp repeats are indicated.

To ask whether breaks can be detected in the *VSG* BESs and whether RAD51 loss impacts on the level and distribution of such breaks, BLISS reads were mapped to all annotated BESs (Fig. 5*A*). This mapping revealed three things. First, BLISS signal was strikingly higher in active BES1 than all silent BESs. Second, BLISS accumulation was most pronounced proximal to the telomere, and particularly at a relatively discrete location around the 3′ end of the *VSG221* gene. Third, the incidence of this most pronounced *VSG221-*associated BLISS signal increased in the *rad51–/*– mutants compared with WT. Quantification of the BLISS signals illustrates these findings. The most prominent, *VSG221*-localised BLISS peak in BES1 had ~30× higher BLISS signal in WT cells and ~197× higher in the *rad51*–/*–* mutant when compared with the average BLISS signal from all inactive BESs at the same location (Fig. 5*A*). In addition, whereas this main BES1 BLISS peak was ranked 119th in terms of BLISS signal in WT cells, it was ranked as 4th highest in the *rad51–/*– mutant (*SI Appendix*, Fig. S6*D*, surpassed only by three mitochondrial maxicircle peaks). These data indicate the presence of a putative DSB or multiple DSBs in the telomere-proximal end of the active *VSG* BES, suggesting an association with active transcription, and that the repair of such breaks requires RAD51.

To understand whether the BLISS read accumulation in BES1 at the 3′ end of *VSG221* represents a discrete DSB, the arrangement of reads mapped to the telomere-proximal region of BES1 in WT and *rad51*–/*–* cells was examined (Fig. 5*B*). In BLISS, DSBs are processed to generate blunt ends for adapter ligation, and a minimally processed, discrete DSB results in forward- and reverse-mapping reads representing downstream and upstream flanks of the break, respectively (Fig. 5*B*) (80, 81). BLISS mapping was not consistent with a discrete DSB at the 3′ end of *VSG221*, since the pronounced BLISS signal in this location was derived almost exclusively from forward-mapping reads, suggesting that they represent the downstream end of a processed DSB. Equivalent levels of reverse mapping reads, representing an upstream end of a DSB, were not detectable in the BES in either WT or *rad51*–/*–* cells, other than a smaller BLISS peak between a short array of 70-bp repeats and a *VSG* pseudogene. In addition, and consistent with the BLISS metaplots (Fig. 5*A*), there was no clear evidence of BLISS reads mapping within the 70-bp repeats in BES1.

Many *VSG* genes contain a conserved 14-mer sequence within their 3′ UTR (20). Though the predominant BLISS signal was upstream of the *VSG221* 14-mer in BES1, enrichment centred on the 14-mer was apparent and became more abundant after loss of RAD51 (*SI Appendix*, Fig. S8*A*). Since we saw R-loop accumulation across the *VSG* archive (*SI Appendix*, Fig. S5), we asked whether the 14-mer might represent a sequence-specific target for break generation common to most *VSG*s. *SI Appendix*, Fig. S8*B* shows metaplots of BLISS and DRIP-seq reads mapped around the 14-mer of all silent *VSG*s. DRIP-seq reads displayed modest accumulation around the 14-mer in WT cells, which became elevated in *RNase H1*–/*–* mutants and depleted in *rad51*–/*–* mutants, consistent with localized accumulation of R-loops. BLISS reads also displayed accumulation that correlated with the DRIP signal in WT cells, perhaps indicating sites of break formation, but the BLISS signal was not increased in *rad51*–/*–* mutants, suggesting that any such breaks are not acted upon by the recombinase across the silent *VSG* archive. Hence, pronounced DNA break accumulation after loss of RAD51 is limited to the single actively transcribed *VSG*.

## Discussion

Immune evasion by antigenic variation in *T. brucei* is driven by locus-specific, RAD51-directed HR of *VSG* genes, but how the reaction is targeted and triggered has been the subject of debate (33). Here, we reveal that RAD51 plays a role in repairing DNA breaks localised to the single transcribed *VSG*, and that such a role involves the recruitment of RNA–DNA hybrids. Our data also suggest that *VSG* switching is a specialised example of *T. brucei* RAD51 functioning genome-wide to localise RNA–DNA hybrids to DNA breaks.

Rad51 from mammals and yeast binds RNA–DNA hybrids in vivo and in vitro (71, 82). Here, DRIP-MS and DRIP-western blot data indicate that *T. brucei* RAD51 also interacts with R-loops, either directly or indirectly. Reduction of S9.6 nuclear signal in *rad51*–/*–* mutants indicates that loss of the recombinase has widespread effects on R-loop homeostasis, which is consistent with a global association between BLISS-predicted DNA breaks and sites of R-loop accumulation, an association that is impaired by loss of RAD51. Hence, this work suggests that *T. brucei* RAD51 promotes R-loop localisation to DNA breaks at many loci, with the implication that RNA–DNA hybrids have so-far unexplored roles in *T. brucei* RAD51-directed DNA break repair. This suggestion is consistent with growing evidence of R-loop activities during DNA break repair in other organisms (57–59).

**Fig. 6.**
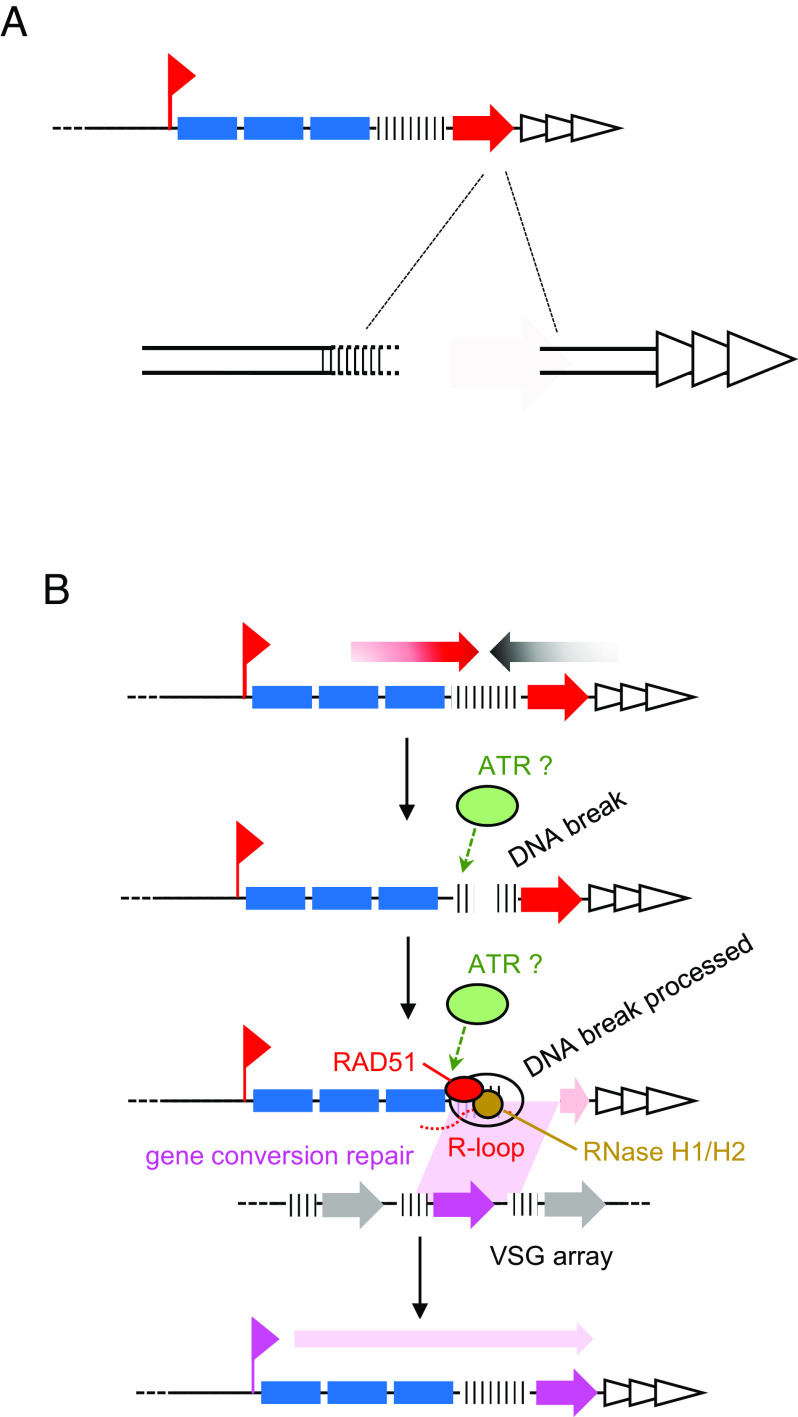
A model for the interplay of R-loops and DNA breakage during *T. brucei* *VSG* switching by recombination. (*A*) A schematic showing the BLISS-predicted location of a DNA DSB in the active *VSG* within the larger expression site. The downstream end of the break is relatively discrete, while the upstream end is more variable (dotted lines) and is predicted to reside in the 70-bp repeats. (*B*) A predicted order of events during *VSG* recombination: A DSB forms within the BES; the DSB is processed and RAD51 is recruited, directing the localisation of R-loops to the break; RAD51 (and perhaps R-loops) catalyses DSB repair via gene conversion or break-induced replication using a silent *VSG* as template. RNase H1 and RNase H2 resolve R-loops once they form. ATR kinase recognises the DNA break and/or the R-loops.

The clearest and strongest association we detect between RNA–DNA hybrids and DNA breaks during RAD51-directed repair in *T. brucei* is in the actively transcribed *VSG* expression site (BES1), where BLISS mapping predicts the most abundant DNA break anywhere in the nuclear genome in *rad51–/*– mutants. These new data allow us to develop an emerging model for how R-loops act during *VSG* switching (Fig. 6). Previously, we postulated that R-loops are the trigger for DNA breaks that lead to *VSG* switching (38, 39). Here, we show that loss of RAD51 has two effects: reduced levels of DRIP-seq signal across the 70-bp repeats of active and inactive BESs and increased levels of *VSG*-localised BLISS signal in the active BES, but not inactive BESs. Hence, rather than R-loops leading to DNA damage, these data suggest that DNA breaks arise in the active BES and lead to RAD51-dependent accumulation of R-loops. In this scenario, it may be that RAD51 promotes *trans* R-loops during the break repair process and RNase H enzymes aid in their resolution, explaining the change in R-loop levels and distribution we see in the silent BESs and in the silent *VSG* arrays after loss of the RAD51, RNase H1 or H2 (38, 39). However, uncertainty about how and where breaks arise in the BES—discussed below—means the 70-bp repeats could be both the cause of damage and a participant in repair. Nonetheless, this model is consistent with data in other organisms suggesting transcribed loci are a focus for DNA repair, including HR (36, 83–86).

Several aspects of this model remain uncertain and are potentially trypanosome-specific. First, what is the nature of the DNA break(s) in the active BES, and where do they form? Though BLISS mapping reveals predominant signal accumulation around the 3′ end of the *VSG* in the active BES, the data do not support the idea that the signal here represents a discrete DSB. Instead, the predominant signal appears to correspond with the downstream end of a DNA break, with no such discrete upstream end (Fig. 6*A*). One explanation for these data, consistent with loss of R-loops in the 70-bp repeats in *rad51–/*– mutants, is that breaks originate in the 70-bp repeats and are processed extensively, but such processing terminates at a more clearly defined region downstream of the *VSG* than upstream in the BES. Alternatively, breaks may form at the 3′ end of the *VSG* and processing occurs more extensively upstream than downstream. However, since BLISS maps breaks in a population, the data could also be explained by variation in DSB localisation with the active BES in different cells, with greater variability in their upstream location and less downstream. A complication in any such scenario is that BLISS mapping involves DSB processing, and so we cannot yet say if such putative diffuse breaks arise during BLISS sample preparation (72), or if extensive DSB end-processing occurs naturally in preparation for recombinational repair during *VSG* switching (Fig. 6*B*). For similar reasons, we cannot be sure if BLISS has mapped a DSB or a distinct form of lesion, such as a stalled replication fork (Fig. 6*B*), which is converted to a DSB during BLISS. Irrespective, the BLISS mapping appears consistent with previous attempts to detect DSBs in the BES by ligation-mediated PCR, which also appeared not to reveal a discrete break location (27, 28, 34, 36).

Questions also arise from this model regarding the dynamics and potential role of the R-loops in *VSG* switching: Why does loss of either characterised nuclear RNase H enzyme increase *VSG* switching (38, 40), and are the hybrids active participants in RAD51 break repair (Fig. 6*B*)? Increased levels of R-loops in *RNase H1*–/*–* mutants is most pronounced in the 70-bp repeats but is also seen across both the active and silent BESs, as well as in silent array *VSG*s. Similarly, although elevated levels of BLISS signal in *rad51*–/*–* mutants is limited to the active BES, loss of the recombinase also changes R-loop levels and patterns in the active and silent BESs, and around silent array *VSGs*. It seems likely that increased DRIP-seq signal in the active BES after RNase H1 loss is consistent with its well-documented roles in removing hybrids (45), including at a DSB at an actively transcribed locus (87, 88). What is less clear is why elevated levels of R-loops at a BES break in RNase H1 mutants might increase *VSG* switching (39), since some evidence suggests increased R-loop levels impede HR (89). However, other studies have suggested R-loop levels may alter DSB end-processing and Rad51 loading (90–96), which may affect repair pathway choice (97). In *T. brucei*, how DNA breaks that lead to *VSG* switching are processed is especially unclear, since mutation of the MRE11–RAD50–NBS1/XRS2 complex does not appear to alter *VSG* switch rate (98), but clearly changes *VSG* donor selection after switch induction by a targeted DSB (64). Impairing BES break repair by RAD51-HR may also open other routes to *VSG* switching, such as microhomology-mediated end joining (87, 88, 99). Increased levels of R-loops in the BES *ESAG*s after RAD51 loss may stem from more a persistent DSB(s) at the *VSG*, resulting in increased stalling of upstream transcription (100–102). Such stalling would increase R-loop levels and may cascade to increased transcriptional switching, resulting in R-loop formation in the previously silent BESs (40). However, changes in DRIP-seq levels in *rad51–/*– and *RNase H1–/*– mutants in the 70-bp repeats of the silent BES may suggest a more active role for R-loops in HR during *VSG* switching. This suggestion may be consistent with alterations in R-loops across the silent subtelomeric *VSG*s. In other eukaryotes, several studies implicate R-loops in promoting break repair (103–106), including evidence for the RNA providing an active role in strand exchange (83). It is therefore possible that the changing patterns of DRIP-seq signal, we see in *rad51*–/*–* and *RNase H1*–/*–* mutants in the silent BES and array *VSG*s reflect, respectively, decreased and increased formation of R-loops in these loci as a result of impaired or enhanced RAD51-directed homology search and strand exchange during *VSG* recombination. Thus, RAD51-directed *trans* R-loops may be widespread in *T. brucei*.

Unlike the actively transcribed BES, the function and formation of R-loops at centromeres and at TSSs is unclear, including why they might be acted upon by RAD51, since neither sequence is a clear site of BLISS-mapped DNA breaks. Nonetheless, the damage signalling kinase ATR acts on R-loops in centromeres (107), and centromere-specific histones (108) modulate levels of centromeric R-loops to limit variation, including genome rearrangement and aneuploidy. Moreover, loss of RAD51 undermines centromere-directed DNA replication initiation in *Leishmania* (109). At TSSs, we see considerable overlap in R-loop localisation in the *rad51^–/^*^–^ mutants relative to H2A.Z localisation in WT cells. Although we cannot currently say what this interrelated mapping reveals, we have previously documented accumulation of DNA damage at *T. brucei* TSSs after loss of RNase H2A (38), and H2A.Z has been implicated in DNA repair (110, 111) and replication (112).

Our data do not reveal a role for RAD51 in TERRA association with telomeres in *T. brucei*. Though this observation differs from mammals and yeast (70, 71), it may be because Rad51-directed R-loop formation using TERRA occurs when telomeres are short and use homology-directed repair for maintenance. Previous work has shown that critically short telomeres in *T. brucei* are associated with increased *VSG* switching (37, 113, 114), but R-loop levels and location—or RAD51 involvement—have not been tested. Loss of shelterin components in *T. brucei* has also been shown to activate *VSG* switching (28, 115, 116), as has loss of ORC1/CDC6 (117), which in mammals associates with TERRA (118). It is conceivable that TERRA, acted upon by RAD51, provides an alternative route for *VSG* switching to that we describe here, but where R-loops are also involved.

## Materials and Methods

For details of *T. brucei* growth and manipulation, microscopy, and long-read genome assembly, see *SI Appendix, SI Materials and Methods*. DRIP-seq and BLISS analyses were performed essentially as described in refs. 66 and 72, respectively, but full method descriptions are in *SI Appendix, SI Materials and Methods*.

## Supplementary Material

Appendix 01 (PDF)

## Data Availability

BLISS and DRIP-seq sequences are available at the NCBI Sequence Read Archive (SRA) under project number PRJEB61713 (119). Nanopore and Illumina reads used for genome assembly have been deposited to the NCBI SRA under project number PRJNA962304 (120). DRIP-MS data and availability is described Girasol et al., BIORXIV/2023/540366.
