# Supplementary material for: RAD51-mediated R-loop formation acts to repair transcription-associated DNA breaks driving antigenic variation in Trypanosoma brucei
Source: Proc Natl Acad Sci U S A. 2023 Nov 21;120(48):e2309306120. doi: 10.1073/pnas.2309306120 (PMC10691351; doi:10.1073/pnas.2309306120)
Supplement: Supplementary file 1 — Appendix 01 (PDF) [file pnas.2309306120.sapp.pdf]

RAD51-mediated R-loop formation acts to repair transcription-associated DNA breaks driving antigenic variation in *Trypanosoma brucei*

Mark John Girasol[1,2], Marija Krasilnikova[1], Catarina A. Marques[1], Jeziel D. Damasceno[1], Craig Lapsley[1], Leandro Lemgruber[1], Richard Burchmore[1], Dario Beraldi[1], Ross Carruthers[4], Emma M. Briggs[1,3*], Richard McCulloch[1*]

Supporting Information Appendix

SI Materials and Methods

Trypanosome growth and genetic manipulation

Trypanosoma brucei MiTat1.2 (Lister 427) BSF cells and a T7 polymerase/Cas9 expressing derivative line (derived using plasmid pJ1339)(1) were cultured in HMI-9 medium (Life technologies), supplemented with 10% foetal calf serum at 37 ºC with 5% CO_2. For epitope tagging and allele knockout, primers were designed with LeishGEdit (http://www.leishgedit.net/) and donor sequences were PCR-amplified from pPOT plasmids (containing mNeonGreen and blasticidin resistance genes) as previously described[64], along with a single guide RNA. Mutant lines were generated by Amaxa transfection with ethanol-purified PCR products and drug-section to generate clonal lines, which were confirmed by PCR. BES-localised *VSG* genes used for expression analysis by RT-qPCR were as follows(2): VSG221 (427-2, MITat1.2), VSG121 (427-6, MITat1.6), VSG800 (427-18, MITat1.18), VSGT3 (427-21, MITat1.21), VSG13 (427-13, MITat1.13) and VSG224 (427-3, MITat1.3).

Microscopy

For analysis of DNA damage γH2A (1:1,000) antiserum was used, and DNA-RNA hybrids were detected using S9.6 (Kerafast, 1:1,000). To perform immunofluorescence, formaldehyde-fixed parasites were permeabilised with 0.1% IGEPAL and blocked with 1% bovine serum albumin (BSA) for α-γH2A staining. For S9.6 staining, methanol-fixed parasites were instead permeabilised with 0.5% v/v Triton X-100 and blocked with 0.1% w/v BSA plus 0.01% v/v Tween-20 at 37 ºC. Alexa Fluor® Plus 488 secondary antibodies were diluted 1:1,000 in the respective blocking solutions before mounting with 5 µL DAPI Fluoromount-G® (Southern Biotech) and imaging. VSG immunofluorescence analysis was performed following the protocol of Glover et al (2016)(3). Briefly, formaldehyde fixed parasites were adhered to glass slides and blocked with 50% FCS in PBS for 45 min before staining with primary anti-VSG (1:10,000) and secondary Alexa Fluor (1:1,000) antibodies and mounting with DAPI Fluoromount-G® (Southern Biotech). Imaging was performed with an Axioscope 2 fluorescence microscope (Zeiss) 63x/1.40 oil objective, or Leica DiM8 microscope to

acquire 25 Z-stacks across a region of 5 µM thickness. To quantify fluorescence intensity of S9.6 signal, images were obtained using the same exposure times and were later processed on ImageJ (http://imagej.net/) using the same parameters. To assess the efficiency of VSG switching, we used anti-VSG immunofluorescence to determine what proportion of cells expressing VSG221 (normally actively expressed) or VSG121 (normally silent), or both

DRIP-qPCR and DRIP-seq

ChIP-IT® Express Enzymatic (Active Motif, Cat#53035) kit was used for DRIP, as previously described[68]. Briefly, 2 x 10^8 mid-logarithmic phase cells were fixed with 1% formaldehyde before nuclei were isolated and chromatin digested with an enzymatic cocktail. 1 µg chromatin was used for immunoprecipitation (IP) reactions in 100 µL aliquots with 5 µg S9.6 antibody (Kerafast), as per the kit protocol. On-bead RNase H treatment control was performed for each reaction: washed beads were incubated with 300 µL 1X RNase H Buffer (NEB) plus 30 units of RNase H (NEB) or 15 µL nuclease-free water at 37ºC for 3 hr. After chromatin was eluted and reverse crosslinked, DNA was eluted as per the kit protocol. For DRIP-qPCR, eluted DNA and the respective input samples were diluted to 1:10 and 1:100, respectively, before 1 µL was added to a qPCR reaction mix containing 1X Fast SYBR Green Master Mix (Invitrogen) and 400 nM of each forward and reverse qPCR primers. qPCR was performed with a 7500 Real-Time PCR system (Applied Biosystems) as follows: 50 ºC for 2 min and 95 ºC for 2 min, followed by 40 cycles of 95 ºC for 15 sec, 59 ºC for 15 sec and 72 °C for 1 min. Fluorescence intensity was measured at the end of the extension step (72 ºC for 1 min). Analysis was done by first adjusting the CT values according to the dilution factor, i.e., -log2(dilution 100 factor). Subsequently, the adjusted CT values of the DRIP samples and the inputs were compared. For DRIP-seq, DNA libraries were prepared from the chromatin using a Qiagen FX 96 Library Kit (QIAGEN) for sequencing using the Illumina NextSeq 500 platform, to a depth of 15 million 100-bp paired-end reads per sample.

Long-read de novo genome assembly

For long-read assembly, DNA of *Trypanosoma brucei* MiTat1.2 (Lister 427) BSF cells was extracted using MagAttract HMW DNA kit (Qiagen) and sequenced using an Oxford Nanopore Technologies (ONT) MinION device with R9.4.1 chemistry. Basecalling was done using guppy (Linux CPU version 3.3.3). A total of 319075 reads (N50 28.46 kb) were generated and subsequently used for genome assembly using Canu (default settings, predicted genome size = 35 Mb). The resulting assembly was polished with four iterations of Pilon using paired-end (2x75bp) Illumina data from the same strain of parasites.

DRIP-seq data analysis

Analysis was performed as previously described (4). Briefly, TrimGalore was used to trim reads (Phred Score <20) before mapping to either the *T. brucei* Lister 427 v10 (5) or Oxford Nanopore assembly genome (this work) using Bowtie2 in "very-sensitive" mode. Mapped reads with MapQ value < 1 were filtered using

SAMtools and the fold-change between the read depths of DRIP IP DNA relative to input control was calculated in 50-bp bins using DeepTools bamCompare tool, where background was separated from DRIP signals using signal extraction scaling. deepTools computeMatrix, plotProfile, and plotHeatmap functions were used to generate plots.

Breaks labelling in situ and sequencing (BLISS)

BLISS was performed by adapting the the protocol developed by Bouwman et al(6), summarised here. Cells were fixed in 2% methanol-free formaldehyde for 10 min at room temperature, before quenching with 125 µM glycine. 1 x 10^8 cross-linked cells were then lysed using 200 µL lysis buffer 1 (10 mM Tris-HCl, 10 mM NaCl, 1 mM EDTA, 0.2% Triton X-100, pH 8 at 4 ºC) and resuspended in 200 µL of pre-warmed lysis buffer 2 (10 mM Tris-HCl, 150 mM NaCl, 1 mM EDTA, 0.3% SDS, pH 8 at RT). DSBs were blunted in situ using the Quick Blunting Kit (NEB) and BLISS linkers were ligated overnight at 16 ºC using T4 DNA ligase (ThermoFisher). The nuclei were washed with 200 µL 1X CutSmart Buffer (NEB) supplemented with 0.1% Triton X-100 to remove un-ligated linkers before being resuspended in TAIL buffer (10 mM Tris, 100 mM NaCl, 50 mM EDTA, 1% SDS, pH 7.5 at RT), with added Proteinase K (NEB). Samples were incubated, shaking overnight at 55 ºC before DNA was extracted using a phenol/chloroform/isoamyl alcohol (PCI) mixture and ethanol precipitated into 100 µL TE buffer. Sonication with a BioRuptor (30 cycles of 30 s on, 60 s off at high intensity) sheared the DNA which was then concentrated using AMPure XP beads and quantified using Qubit dsDNA BR assay (ThermoFisher Scientific). To prepare libraries, in vitro transcription (IVT) of 100-200 ng of BLISS template gDNA was performed in DNA LoBind tubes using MEGAscript T7 (ThermoFisher) at 37 ºC for 14-15 h (lid temperature set at 70 ºC). The DNA template was subsequently degraded using 2U DNase I (ThermoFisher) and RNA was purified using pre-warmed RNAClean XP beads (Beckman Coulter) at room temperature. Small RNA Seq 3' adapters (Illumina) were ligated using T4 RNA ligase (NEB) at 25 ºC for 2 h, and reverse transcription was performed with SuperScript IV reverse transcriptase (ThermoFisher) at 50 ºC for 50 mins and stopped at 80 ºC for 10 mins. The resultant library was amplified using NEBNext Ultra II Q5 Master Mix by performing 8 cycles initially, then bead-purifying the mixture using 0.8 AMPure XP DNA beads, before further amplification via 4 additional cycles of PCR. Finally, DNA libraries were purified using two-sided AMPure purification and sequenced on an Illumina NextSeq 500 platform to a depth of 60 million single-end reads per sample. Sequencing was done at Glasgow Polyomics.

BLISS reads were trimmed to remove library barcodes and UMIs using cutadapt, allowing up to 1 mismatch, and the library barcode and UMI were added to the read name using a custom python script, cutadaptInfoToFastq.py. Reads were then mapped to the *Trypanosoma brucei* Lister 427 v10 genome using bowtie2 under default settings. The aligned reads were exported in BAM format before sorting with SAMtools and deduplicating using the UMI with umi_tools dedup. This ensured that reads mapping to the same locations with different UMIs come from distinct DSBs rather than being PCR duplicates. Finally, the filtered reads were normalized with bamCoverage and then exported as a bigwig file. The bigwig files were

either visualized as tracks using Integrated Genome Viewer or plotted as signal profiles or heatmaps as described for DRIP-seq.

Read depth peaks were then detected as a proxy for regions of DNA breaks using the macs2 peak caller version 2.2.7.1(7). We first filtered the alignment bam files to retain only reads that could be confidently assigned to a single genomic region by requiring reads to have mapping quality of at least 10 and excluded alignments marked as "supplementary" by the aligner (bwa mem). For peak calling we used "macs2 callpeak" with these settings: effective genome size (-g) 35000000, keep all duplicates (--keep-dup all), do not build model (--nomodel), extend reads by 80 bp (--extsize 80). Other parameters were set to default to select only peaks with FDR < 0.05, and without an input control. The analysis pipeline of the BLISS data is available at https://github.com/glaParaBio/bliss_dna_break_analysis as Snakemake pipeline.

1. F. Rojas *et al.*, Oligopeptide Signaling through TbGPR89 Drives Trypanosome Quorum Sensing. *Cell* **176**, 306-317 e316 (2019).
2. C. Hertz-Fowler *et al.*, Telomeric expression sites are highly conserved in Trypanosoma brucei. *PLoS ONE.* **3**, e3527 (2008).
3. L. Glover, S. Hutchinson, S. Alsford, D. Horn, VEX1 controls the allelic exclusion required for antigenic variation in trypanosomes. *Proceedings of the National Academy of Sciences of the United States of America* **113**, 7225-7230 (2016).
4. E. Briggs, G. Hamilton, K. Crouch, C. Lapsley, R. McCulloch, Genome-wide mapping reveals conserved and diverged R-loop activities in the unusual genetic landscape of the African trypanosome genome. *Nucleic Acids Res* **46**, 11789-11805 (2018).
5. L. S. M. Muller *et al.*, Genome organization and DNA accessibility control antigenic variation in trypanosomes. *Nature* **563**, 121-125 (2018).
6. B. A. M. Bouwman *et al.*, Genome-wide detection of DNA double-strand breaks by in-suspension BLISS. *Nature protocols* **15**, 3894-3941 (2020).
7. Y. Zhang *et al.*, Model-based analysis of ChIP-Seq (MACS). *Genome biology* **9**, R137 (2008).

Supporting figure legends

Figure S1. CRISPR-Cas9 generation and analysis of *T. brucei RAD51* null mutants. (A) Upper agarose gels show PCR of a *rad51*-/- transformant, detailing the expected integration of NEO and BSD resistance cassettes, replacing the *RAD51* ORF; P, parental TbCas9/T7 (‘wild type’) cell line. Lower gel shows a western blot of RAD51 protein expression in the same mutant using an anti-RAD51 antibody; EF1α was used as a loading control. (B) Sample images of S9.6 immunofluorescence, comparing parental TbCas9/T7 cells and *rad51*-/- mutants, with (+) or without (-) *E. coli* RNase HI treatment; scale bar = 10 µm.

Figure S2. Testing DRIP efficacy. (A). Analysis of DRIP-seq data from two independent *rad51*-/- mutants in the presence (grey) or absence (magenta) of *E. coli* RNase HI; graphs depict metaplots of reads mapped to all RNA Pol II–transcribed CDSs (scaled to size), including +/- 1 kb. (B) DRIP-qPCR of *rad51*-/- cells,

quantifying recovery of R-loops from BES1 (*ESAG6*, *ESAG8*, *VSG221*) and BES3 (*VSG121*). (C) DRIP-qPCR comparing wild type and *rad51*-/- R-loop enrichment within the actin CDS. In all DRIP-qPCR analyses, abundance is expressed as a percentage in IP relative to input sample, with or without treatment with *E. coli* RNase HI; error bars represent SEM of two independent replicates.

Figure S3. DRIP-seq signal correlates with H2A.Z enrichment in transcription start sites in *rad51-/-* mutants. (A) Pearson correlation coefficients of DRIP-seq signal in wild-type (WT), *rad51*-/- and *RNase H1*-/- (*RH1*-/-) cells relative to H2A.Z ChIP-seq signal in WT cells across a 40-Kb genomic window surrounding all TSSs. (B) Metaplots of *rad51-/-* DRIP-seq reads along a 40-Kb window surrounding the H2A.Z ChIP-seq peaks at TSSs, showing the difference between samples treated and not treated with *E. coli* RNase HI.

Figure S4. Loss of *T. brucei* RAD51 alters R-loop localisation to centromeres. (A) DRIP-seq read mapping (DRIP/input) across one Nanopore contig covering a complete centromere (black box), comparing *RNase H1-/-* (*rh1-/-*, blue), wild-type (WT, orange) and *rad51*-/- (magenta) cells. (B) Metaplots (top panel) and heatmaps (bottom panel) of wild type, *rad51*-/- and *rh1*-/- DRIP-seq signals (relative to input) across all centromere-containing contigs (scaled to the same size); data shows mean plus SEM (shaded).

Figure S5. Loss of *T. brucei* RAD51 alters R-loop localisation around transcriptionally silent subtelomeric array *VSG*s. (A) DRIP-seq read mapping (DRIP/input) across one Nanopore contig containing an array of subtelomeric *VSG*s, comparing *RNase H1-/-* (*rh1-/-*, blue), wild-type (WT, orange) and *rad51*-/- (magenta) cells. (B) Metaplots (top panel) and heatmaps (bottom panel) of wild type, *rad51*-/- and *rh1*-/- DRIP-seq signals (relative to input; mean is shown and SEM is shaded) across the predicted CDSs of all identified subtelomeric *VSG*s (scaled to the same size), plus 1 kb of upstream and downstream sequences.

Figure S6. Genome-wide analysis of DNA breaks in *T. brucei* BSF cells by BLISS. (A) Distribution of MACS2-predicted peaks of BLISS signal in wild type (WT) and *rad51*-/- cells; proportions of total BLISS peaks residing in different genomic locations are shown; ‘others’ represents RNA Pol II PTUs. (B) Ranking of BLISS peak signal in WT and *rad51-/-* cells; N reads at pileup represents BLISS reads/million reads at the summit of a BLISS peak; BLISS reads mapping to active BES1 are highlighted as a red dot. (C) Mapping of BLISS signal (read count per million mapped reads) across selected genome features (data are shown as average normalised signal across the region of interest); in each case, metaplots depict the mean signal plus SEM (shaded) across all CDSs or centromeres (scaled to the same size), including 1 Kb of upstream and downstream sequence. (D) Metaplots comparing BLISS signal around all predicted TSSs or TSSs (identified as divergent (d) or convergent (c) strand switch regions, SSRs), in wildtype (WT) and *rad51*-/- cells.

Figure S7. DSB and R-loop distribution around tRNA and protein-coding genes. Metaplots showing the distribution of DRIP-seq signal (DRIP/input) and BLISS signal (average normalised signal across the region of interest) around all tRNA genes and all RNA PolII transcribed CDSs in wild type (WT) cells and *rad51*-/- mutants; in each case, metaplots depict the mean signal plus SEM (shaded) across the genes, which are scaled to the same size, and include 1 Kb of upstream and downstream sequence.

Figure S8. DSB and R-loop distribution around the conserved *VSG* 14-mer motif. (A) IGV track showing BLISS signal (read counts per million mapped reads) around the actively expressed *VSG* gene, *VSG221*; arrow denotes the location of the conserved 14-mer sequence. (B) Metaplot showing distribution of DRIP-seq signal (DRIP/input) in sequences centred on the 14-mer at the 3' end of all *VSG* genes, comparing wild type, *rad51*-/- and *RNase H1*-/- cells. (C) Metaplot showing BLISS signal (mean reads per million mapped reads, and SEM shaded) centred on the 14-mer of all *VSG*s, comparing wild type and *rad51*-/- cells.

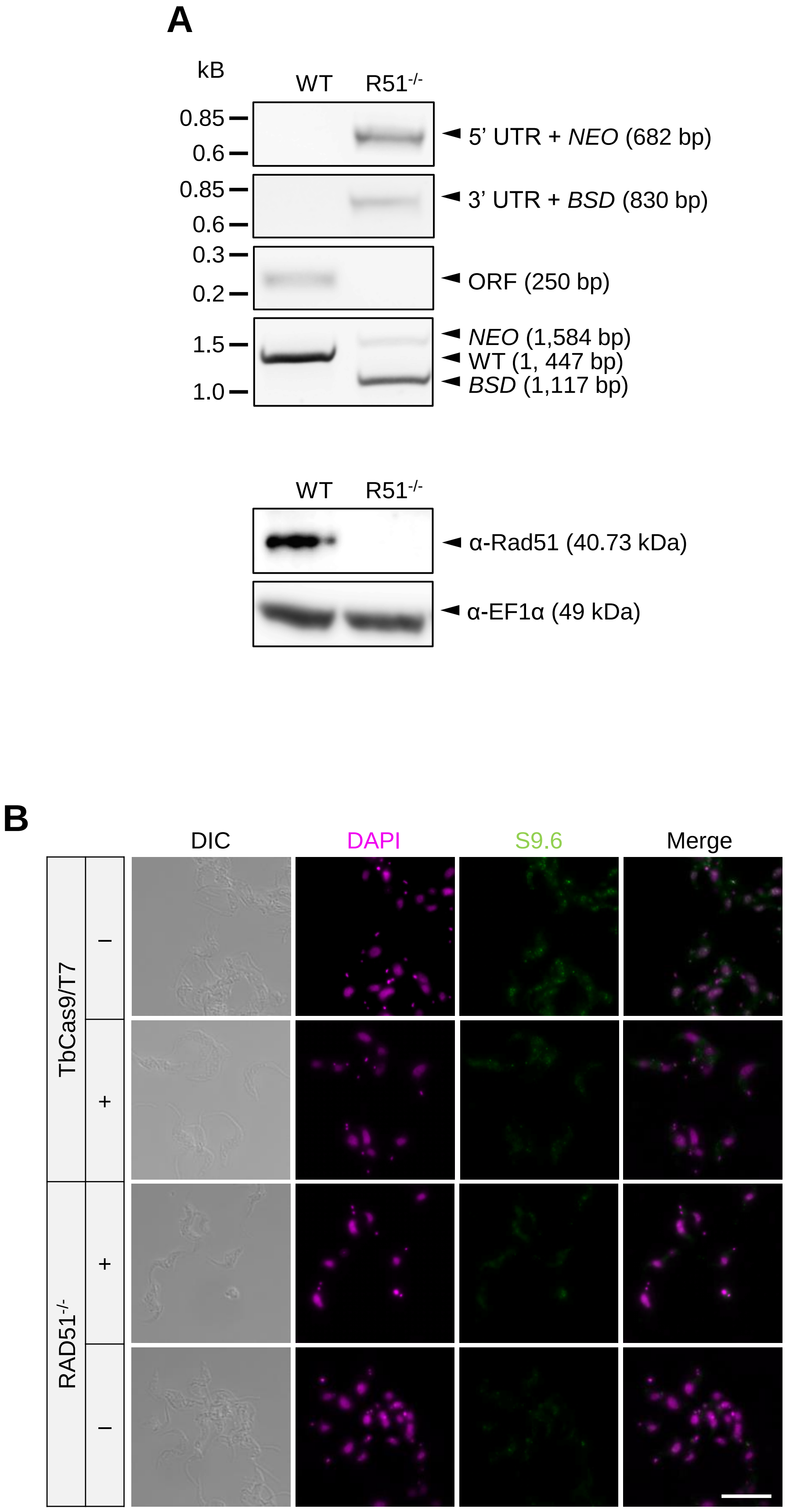
A
kB
WT
R51-/-
0.85
0.6
5' UTR + NEO (682 bp)
0.85
0.6
3' UTR + BSD (830 bp)
0.3
0.2
ORF (250 bp)
1.5
1.0
NEO (1,584 bp)
WT (1, 447 bp)
BSD (1,117 bp)
WT
R51-/-
α-Rad51 (40.73 kDa)
α-EF1α (49 kDa)
B
DIC
DAPI
S9.6
Merge
TbCas9/T7
RAD51-/-
–
+
+
–

Fig S1

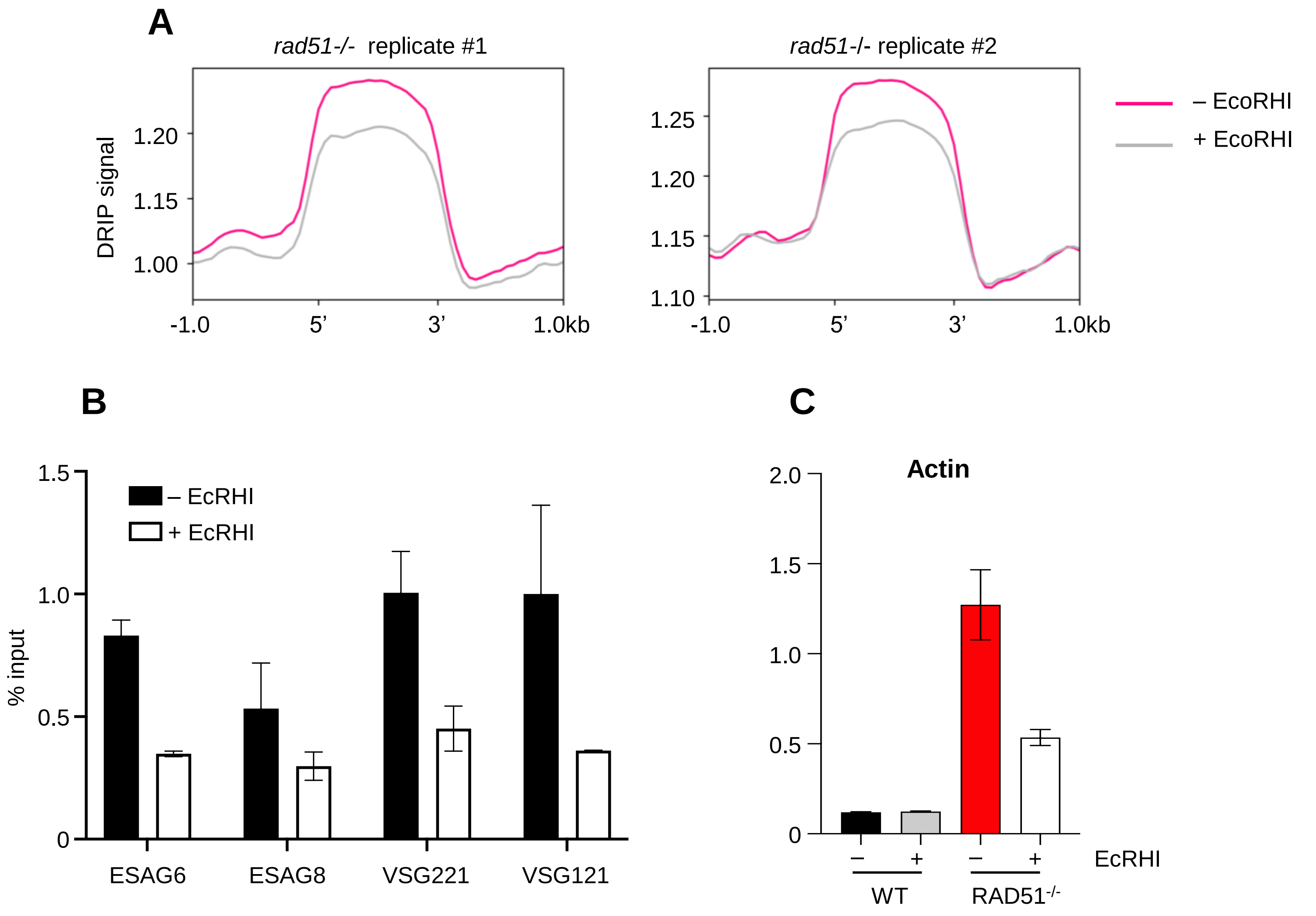
A
rad51-/- replicate #1
rad51-/- replicate #2
DRIP signal
1.20
1.15
1.00
1.25
1.20
1.15
1.10
-1.0
5'
3'
1.0kb
– EcoRHI
+ EcoRHI
B
% input
1.5
1.0
0.5
0
– EcRHI
+ EcRHI
ESAG6
ESAG8
VSG221
VSG121
C
Actin
2.0
1.5
1.0
0.5
0
– + – +
EcRHI
WT
RAD51-/-

Fig S2

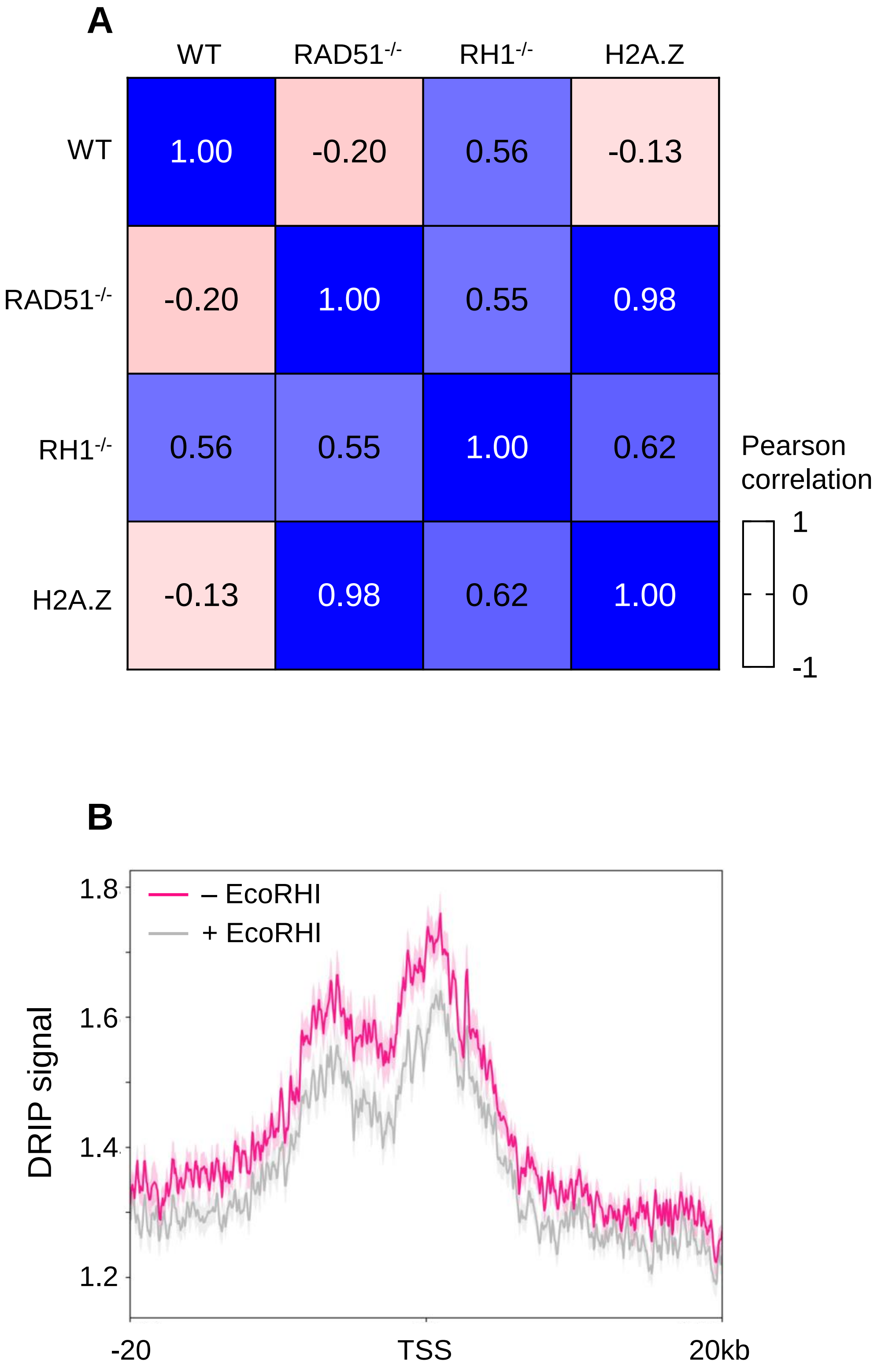
A
WT
RAD51-/-
RH1-/-
H2A.Z
1.00
-0.20
0.56
-0.13
0.55
0.98
0.62
Pearson correlation
1
0
-1
B
– EcoRHI
+ EcoRHI
DRIP signal
1.8
1.6
1.4
1.2
-20
TSS
20kb

Fig S3

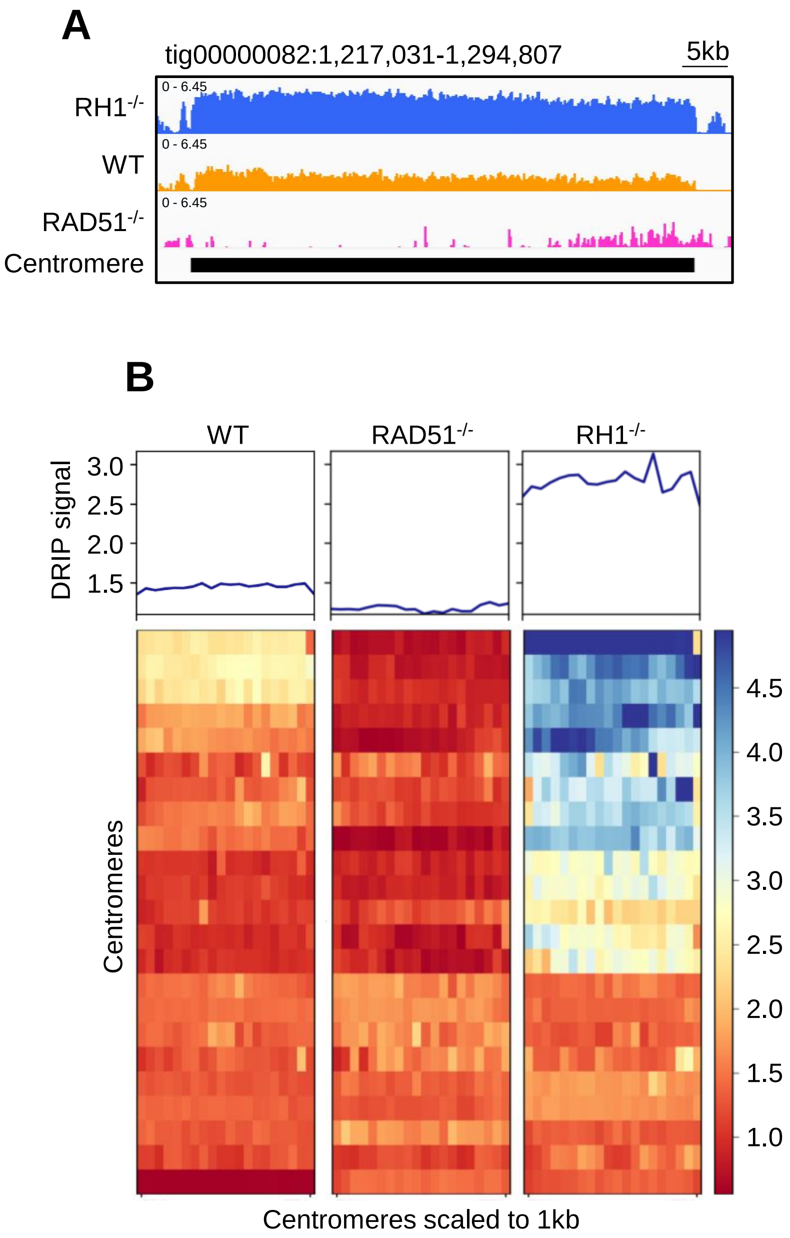
A
tig00000082:1,217,031-1,294,807
5kb
RH1-/-
WT
RAD51-/-
Centromere
0 - 6.45
B
WT
RAD51-/-
RH1-/-
DRIP signal
3.0
2.5
2.0
1.5
Centromeres
4.5
4.0
3.5
3.0
2.5
2.0
1.5
1.0
Centromeres scaled to 1kb

Fig S4

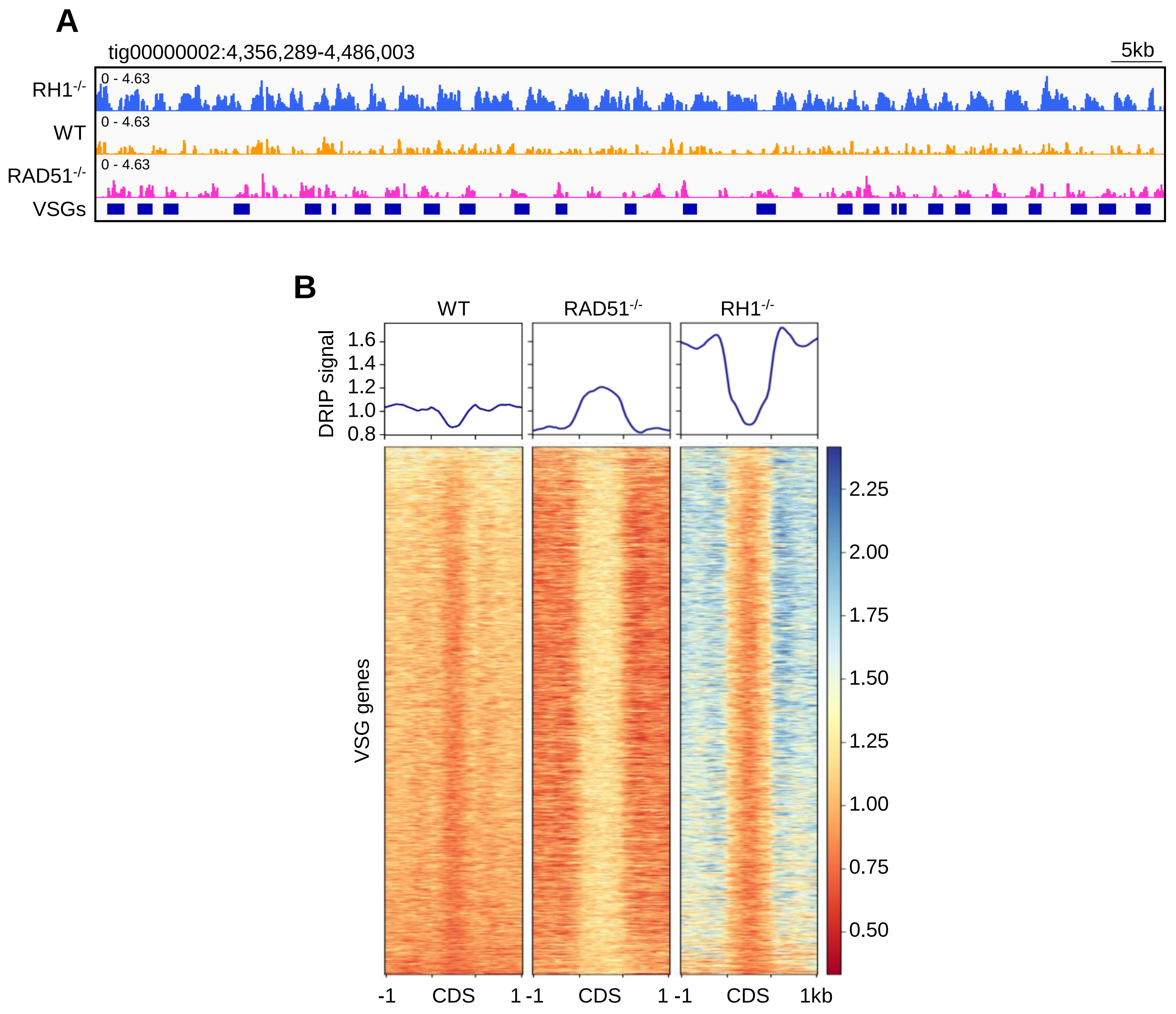
A
tig00000002:4,356,289-4,486,003
5kb
RH1-/-
WT
RAD51-/-
VSGs
0 - 4.63
B
WT
RAD51-/-
RH1-/-
DRIP signal
1.6
1.4
1.2
1.0
0.8
VSG genes
2.25
2.00
1.75
1.50
1.25
1.00
0.75
0.50
-1
CDS
1 -1
CDS
1 -1
CDS
1kb

Fig S5

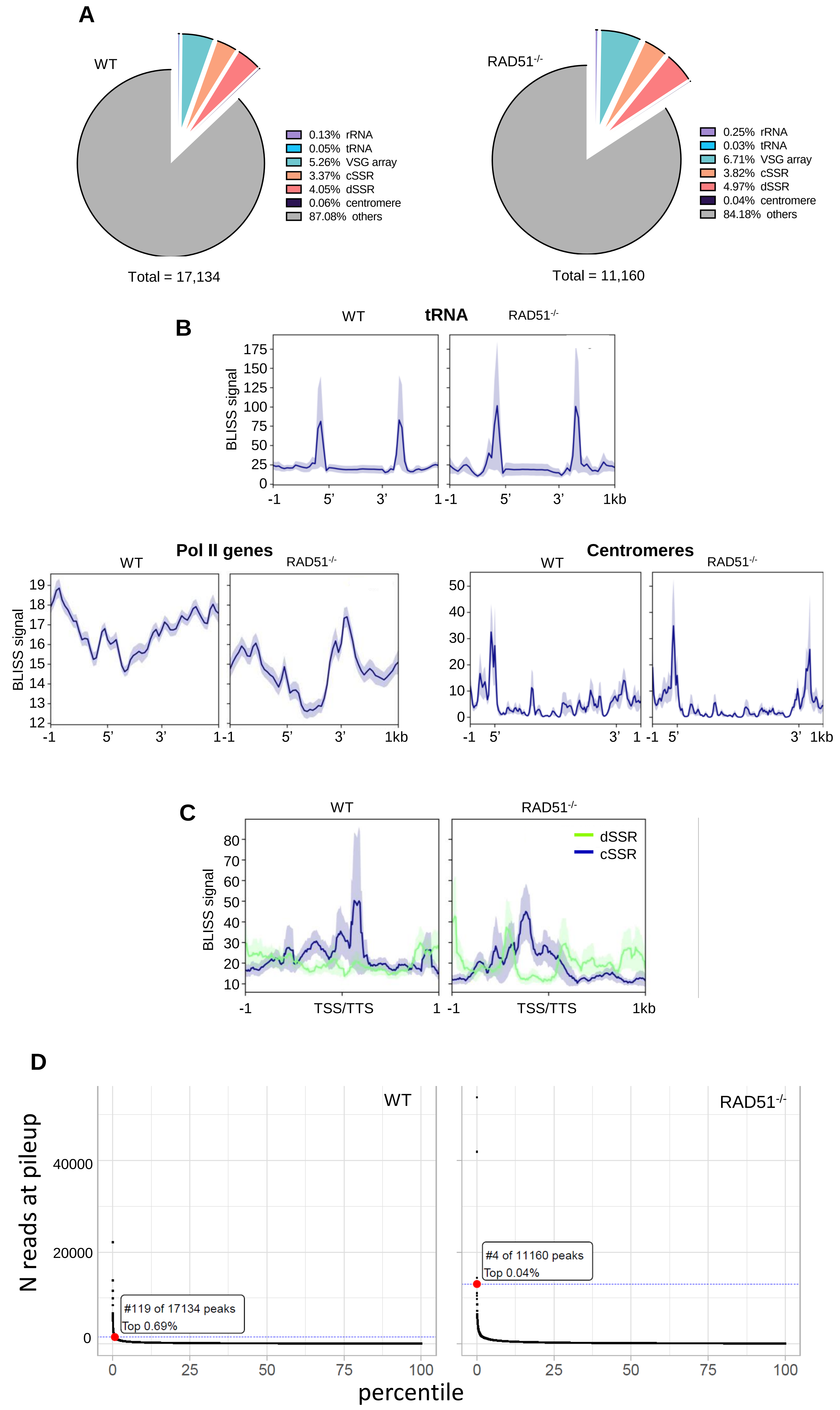

A
WT
0.13% rRNA
0.05% tRNA
5.26% VSG array
3.37% cSSR
4.05% dSSR
0.06% centromere
87.08% others
Total = 17,134
RAD51-/-
0.25% rRNA
0.03% tRNA
6.71% VSG array
3.82% cSSR
4.97% dSSR
0.04% centromere
84.18% others
Total = 11,160
B
tRNA
WT
RAD51-/-
BLISS signal
-1
5'
3'
1kb
Pol II genes
Centromeres
C
dSSR
cSSR
TSS/TTS
D
#119 of 17134 peaks
Top 0.69%
#4 of 11160 peaks
Top 0.04%
N reads at pileup
percentile

Fig S6

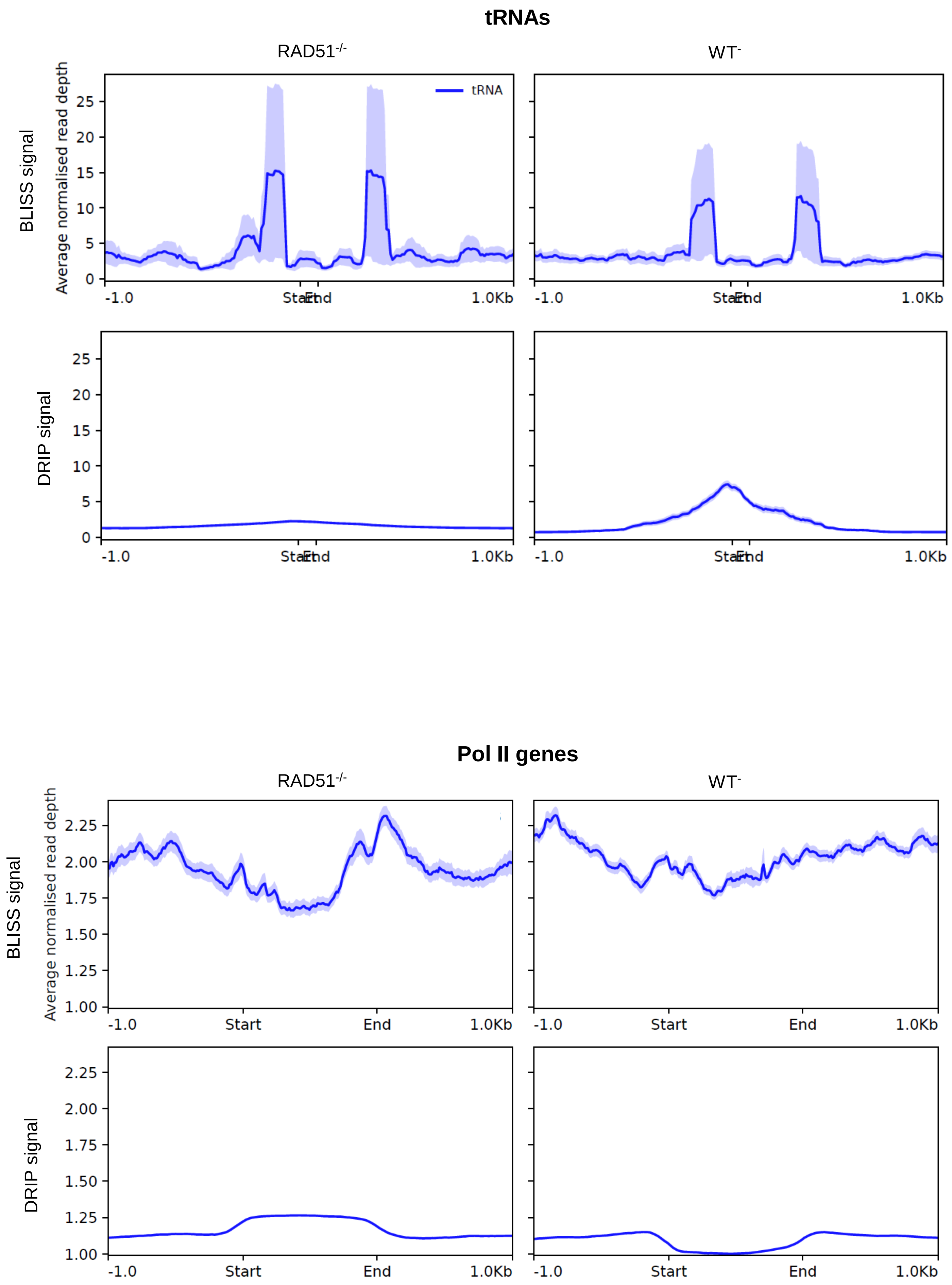

tRNAs
RAD51-/-
WT-
BLISS signal
Average normalised read depth
tRNA
DRIP signal
0
5
10
15
20
25
-1.0
Start
End
1.0Kb
Pol II genes
RAD51-/-
WT-
BLISS signal
Average normalised read depth
DRIP signal
1.00
1.25
1.50
1.75
2.00
2.25
-1.0
Start
End
1.0Kb

Fig S7

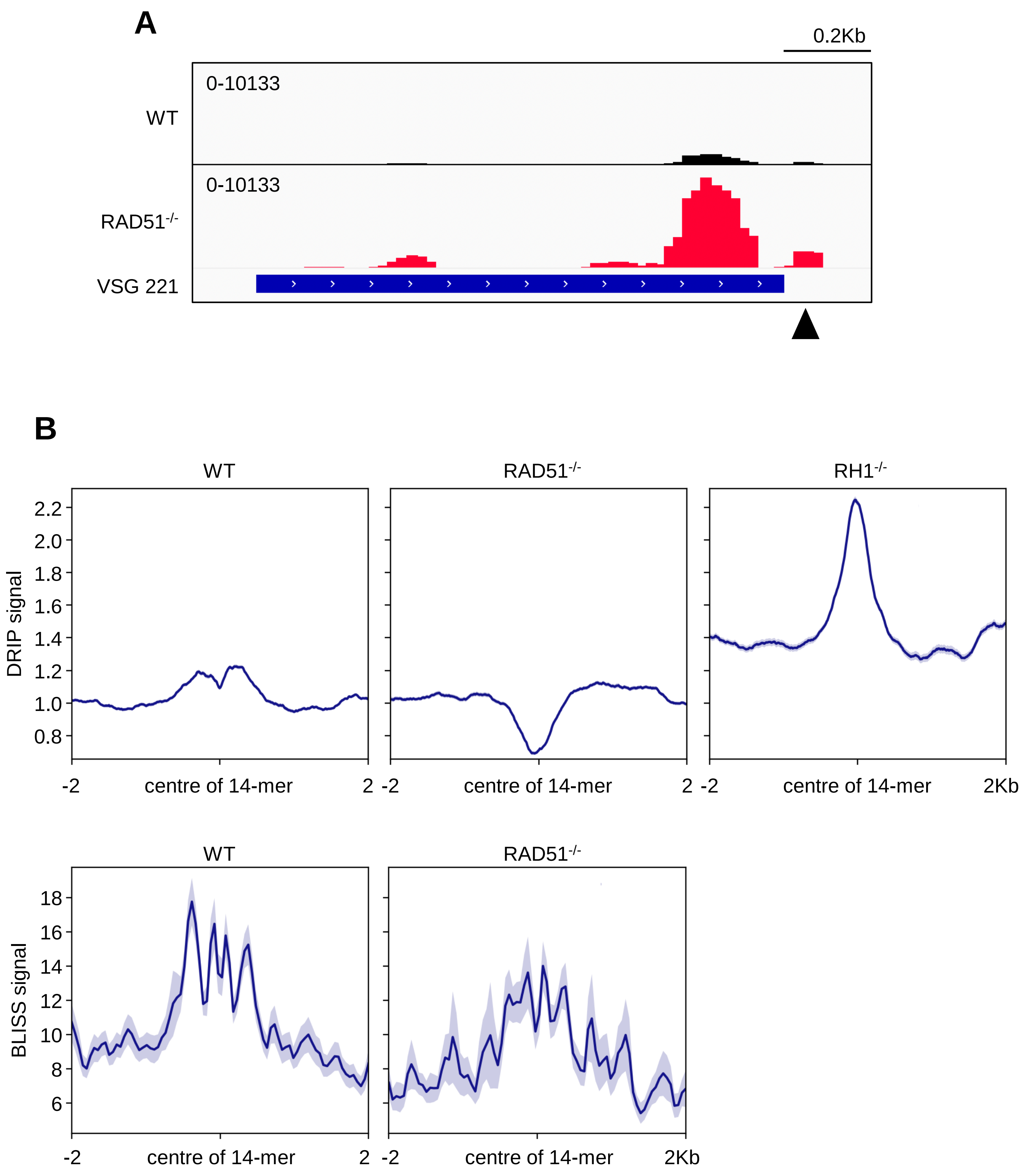
A
0.2Kb
0-10133
WT
0-10133
RAD51-/-
VSG 221
B
WT
RAD51-/-
RH1-/-
DRIP signal
2.2
2.0
1.8
1.6
1.4
1.2
1.0
0.8
-2
centre of 14-mer
2
2Kb
BLISS signal
18
16
14
12
10
8
6

Fig S8
